# Supplementary material for: Synthetic molecular switches driven by DNA-modifying enzymes
Source: Nat Commun. 2024 May 6;15:3781. doi: 10.1038/s41467-024-47742-2 (PMC11074287; doi:10.1038/s41467-024-47742-2)
Supplement: Supplementary file 1 — Supplementary Information [file 41467_2024_47742_MOESM1_ESM.pdf]

Supplementary Information for

Synthetic molecular switches driven by DNA-modifying enzymes

Hong Kang[1,2], Yuexuan Yang[1] & Bryan Wei[1*]
[1] School of Life Sciences, Center for Synthetic and Systems Biology, Tsinghua University, Beijing 100084, China.
[2] Department of Biochemistry and Biophysics, University of Pennsylvania Perelman School of Medicine, Philadelphia, PA, USA.
*Email: bw@tsinghua.edu.cn (B.W.)

This PDF file includes:

Supplementary Figures 1 to 61
Supplementary Table 1
Supplementary References

Other Supplementary Information for this manuscript includes the following:

Supplementary Dataset 1: DNA sequences

Supplementary Figures

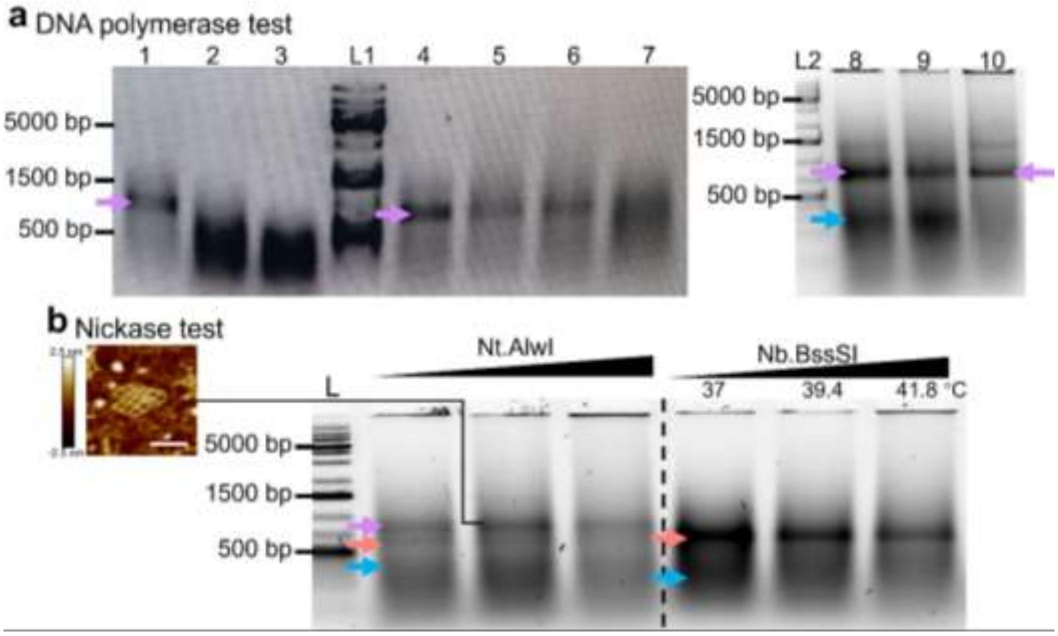

Supplementary Figure 1. Enzymes screening for switch functionality. (a) DNA polymerase screening. Four DNA polymerase enzymes were tested with lattice DNA nanostructures, including *Bst* DNA polymerase large fragments, Phi 29 polymerase, *Bsu* DNA polymerase large fragments and Klenow fragments (3'→5' exo-) (NEB). Lanes L1 and L2: 1 kb+ ladder. Lane 1: DNA nanostructure was formed in 1× TE buffer with *Bst* DNAP enzyme. Lane 2: structure formed with 1× *Bst* DNAP buffer. Lane 3: The structure formed with 1× *Bst* DNAP buffer was mixed with *Bst* DNAP enzyme. Lane 4: Complete structure (purple arrow). Lane 5: the structure was formed in 1× Phi 29 buffer and mixed with Phi 29 enzyme. Lane 6: structure with 1× Phi 29 buffer. Lane 7: the structure with 1× Phi 29 buffer was digested with Phi 29 enzyme. Lane 8: The structure was cut by *Bsu* DNAP into small parts (blue arrow). Lane 9: The structure was cut by Klenow with similar efficiency as *Bsu* DNAP. *Bst* DNAP worked unideally and the rest two didn't work. We selected the *Bsu* DNAP as the working polymerase in this work. (b) Nicking endonuclease selection. We tested Nt.AlwI and Nb.BssSI in separated parts of DNA nanostructures (indicated by pink and blue arrows) to reform a complete structure at different temperatures for 17 h of incubation. Only Nt.AlwI produced the coupling structure imaged under an AFM microscope. Scale bar: 50 nm.

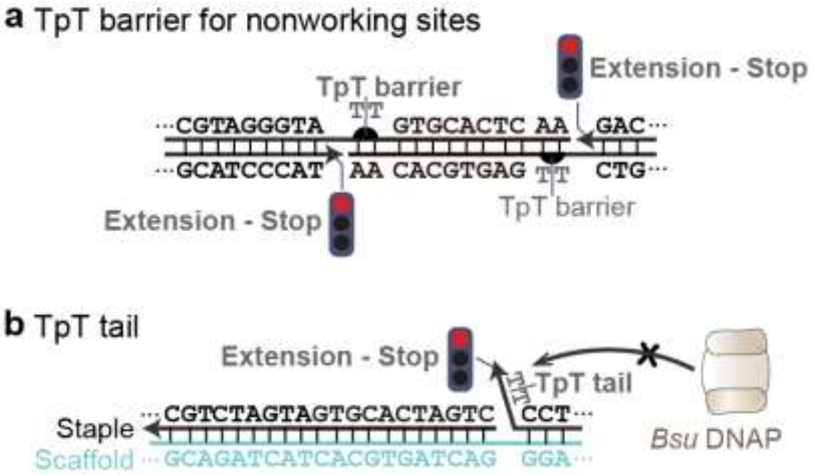

Supplementary Figure 2. Schematics of TpT barrier and TpT tail working mechanism. (a) Schematics of TpT barrier for nonworking sites. The common sticky ends with TpT barriers at both strands disabled them as template synthesized strands to disassociate the coupling partners. (b) Schematic of TpT tail. The TpT tail at the 3' end of the staple prevented *Bsu* DNAP from polymerization with the scaffold as an extension template. Tails with other sequences or lengths would function similarly. TpT was chosen because tails with consecutive dT are known with little negative impact on origami folding.

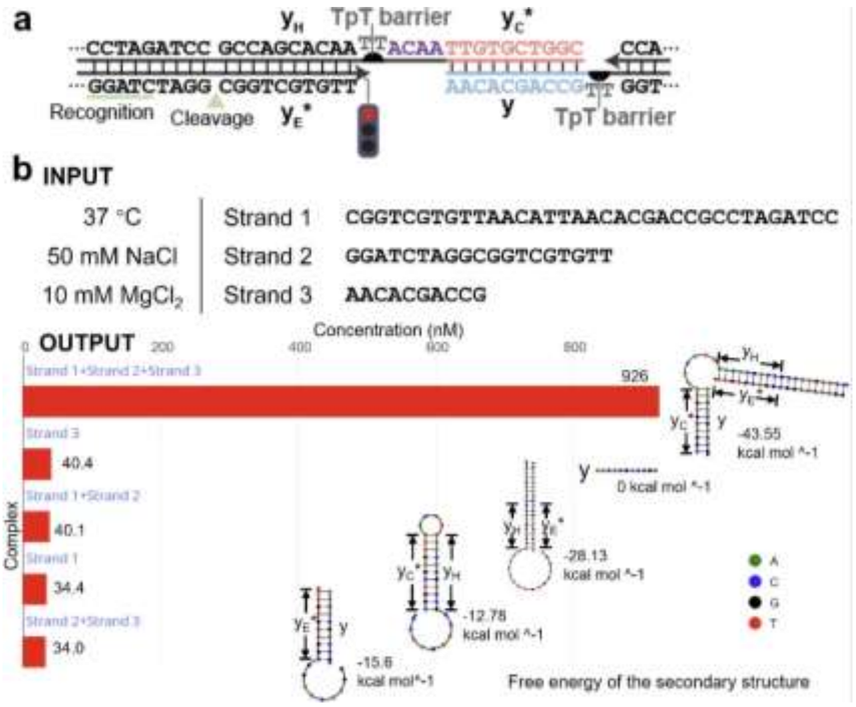

Supplementary Figure 3. Thermodynamic analysis of ON state in Fig. 2b. (a) Schematic of complex Y in switch Y. (b) NUPACK[1] analysis of complex Y. With three entities (yH - yC*, yE*, and y) as the input, simulated results from NUPACK showed the predominant product as the coupled complex rather than the closed hairpin.

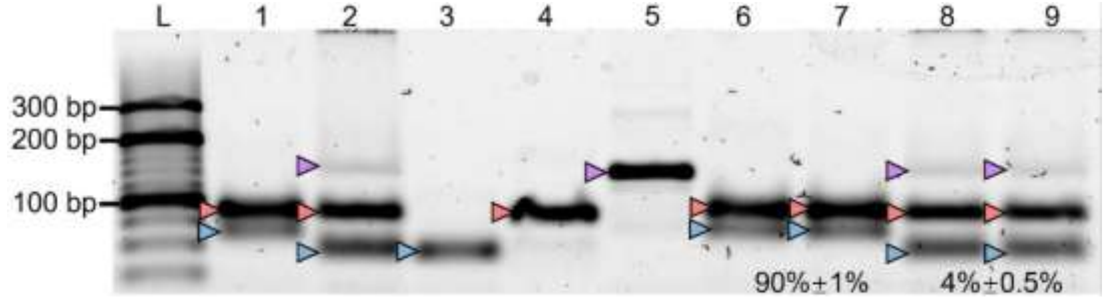

Supplementary Figure 4. Native polyacrylamide gel electrophoresis triplicated results of FX reaction and BX reaction in the single-pair binding system in 2 hours. (Individual reactions proceeded in dedicated pathways, FX only or BX only.) Lane L: 20 bp ladder. Lane 3: T-junction X_{II}. Lane 4: T-junction X_{I}. Lane 5: complex X (formed by two T-junctions X_{I} and X_{II}). Lanes 1, 6 and 7: the product of forward reaction X (FX). Lanes 2, 8 and 9: the product of backward reaction X (BX). The triangles with black edges in red, blue and purple indicate the product bands of X_{I}, X_{II} and X respectively, which apply to the rest Supplementary unless otherwise stated. Numbers at the bottom of the bands indicate the reaction efficiencies (mean ± SD, *N*=3).

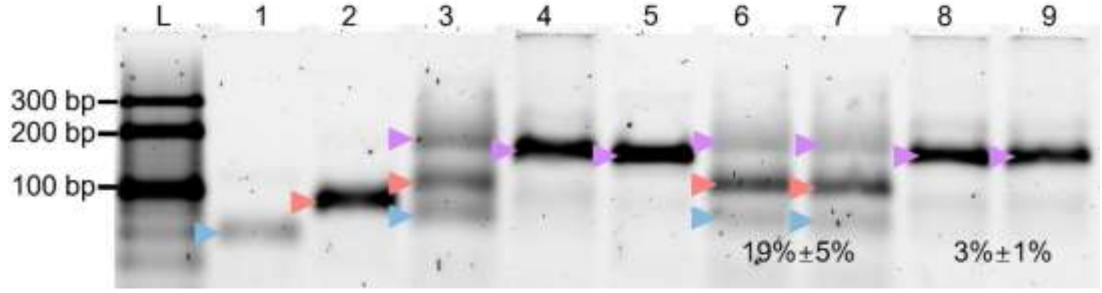

Supplementary Figure 5. Native polyacrylamide gel electrophoresis triplicated results of FY reaction and BY reaction in the single-pair binding system in 2 hours. (Individual reactions proceeded in dedicated pathways, FY only or BY only.) Lane L: 20 bp ladder. Lane 1: T-junction Y_{II}. Lane 2: T-junction Y_{I}. Lane 5: complex Y (formed by two T-junctions Y_{I} and Y_{II}). Lanes 3, 6 and 7: the product of backward reaction Y (BY). Lanes 4, 8 and 9: the product of forward reaction Y (FY); The triangles in red, blue and purple indicate the product bands of Y_{I}, Y_{II} and Y respectively, which apply to the rest Supplementary unless otherwise stated. Numbers at the bottom of the bands indicate the reaction efficiencies (mean ± SD, *N*=3).

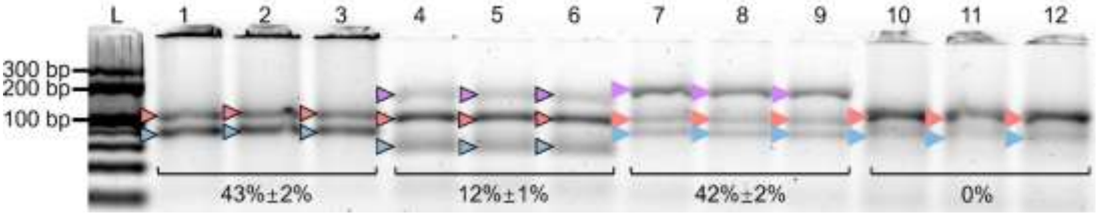

Supplementary Figure 6. Native polyacrylamide gel electrophoresis triplicated results of FX reaction, BX reaction, FY reaction, and BY reaction in the single-pair binding system in 24 hours. (Individual reactions proceeded in dedicated pathways, FX only, BX only, FY only, or BY only.) Lane L: 20 bp ladder. Lanes 1, 2 and 3: the product of FX reaction. Lanes 4, 5 and 6: the product of BX reaction. Lanes 7, 8 and 9: the product of FY reaction. Lanes 10, 11 and 12: the product of BY reaction. Numbers at the bottom of the bands show the reaction efficiencies (mean ± SD, *N*=3).

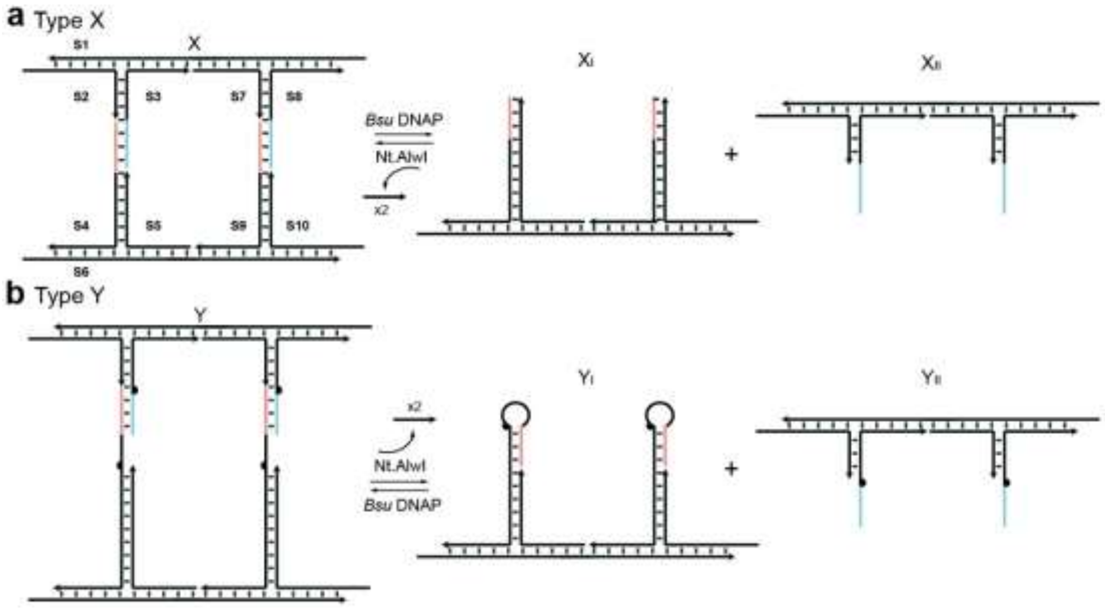

Supplementary Figure 7. Schematics of the dual-pair binding site minimalist system. (a) Type X. The *Bsu* DNA DNAP drives the disassociation and the Nt.AlwI endonuclease enzyme drives the association. (b) Type Y. enzymes in (a) play inverse roles in type Y. The length of the binding site is 16 nt. The black solid semicircles indicate the TpT barriers.

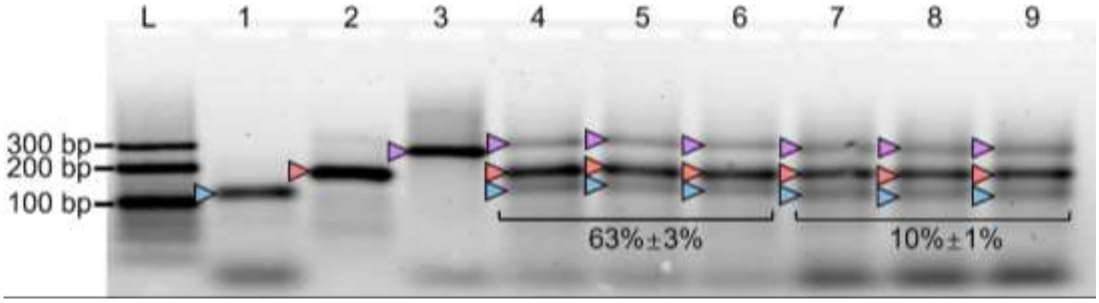

Supplementary Figure 8. Native polyacrylamide gel electrophoresis triplicated results of FX reaction and BX reaction in the dual-pair binding system in 2 hours. (Individual reactions proceeded in dedicated pathways, FX only or BX only.) Lane L: 20 bp ladder. Lane 1: dual-pair binding site unit X_{II}. Lane 2: dual-pair binding site unit X_{I}. Lane 3: dual-pair binding site complex X. Lanes 4, 5 and 6: the product of FX reaction. Lanes 7, 8 and 9: the product of BX reaction. Numbers at the bottom of the bands indicate the reaction efficiencies (mean ± SD, *N*=3).

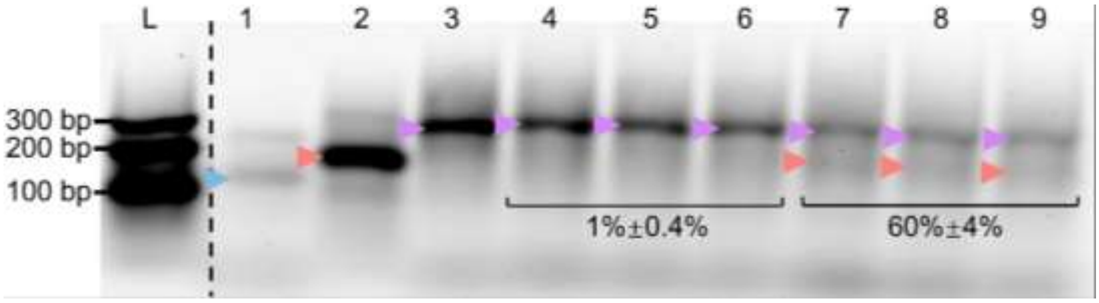

Supplementary Figure 9. Native polyacrylamide gel electrophoresis triplicated results of FY reaction and BY reaction in the dual-pair binding system in 2 hours. (Individual reactions proceeded in dedicated pathways, FY only or BY only.) Lane L: 20 bp ladder. Lane 1: dual-pair binding site unit Y_{II}. Lane 2: dual-pair binding site unit Y_{I}. Lane 3: dual-pair binding site complex Y. Lanes 4, 5 and 6: the product of FY reaction. Lanes 7, 8 and 9: the product of BY reaction. Numbers at the bottom of the bands indicate the reaction efficiencies (mean ± SD, *N*=3).

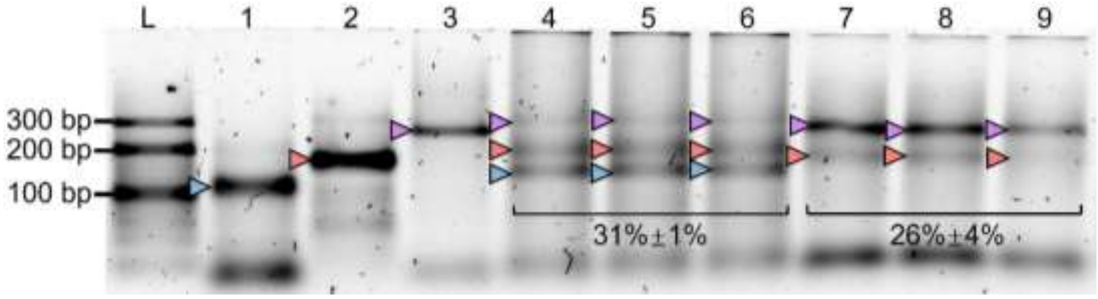

Supplementary Figure 10. Native polyacrylamide gel electrophoresis triplicated results of FX reaction and BX reaction in the dual-pair binding system in 24 hours. (Individual reactions proceeded in dedicated pathways, FX only or BX only.) Lane L: 20 bp ladder. Lane 1: dual-pair binding site unit X_{II}. Lane 2: dual-pair binding site unit X_{I}. Lane 3: dual-pair binding site complex X. Lanes 4, 5 and 6: the product of FX reaction. Lanes 7, 8 and 9: the product of BX reaction. Numbers at the bottom of the bands indicate the reaction efficiencies (mean ± SD, *N*=3).

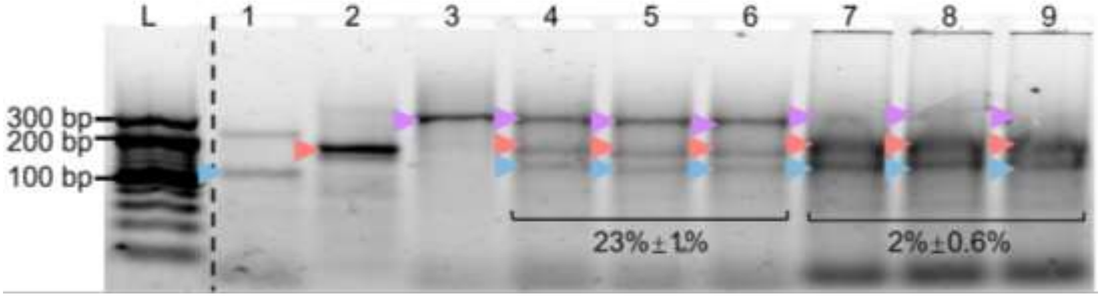

Supplementary Figure 11. Native polyacrylamide gel electrophoresis triplicated results of FY reaction and BY reaction in the dual-pair binding system in 24 hours. (Individual reactions proceeded in dedicated pathways, FY only or BY only.) Lane L: 20 bp ladder. Lane 1: dual-pair binding site unit Y_{II}. Lane 2: dual-pair binding site unit Y_{I}. Lane 3: dual-pair binding site complex Y. Lanes 4, 5 and 6: the product of FY reaction. Lanes 7, 8 and 9: the product of BY reaction. Numbers at the bottom of the bands indicate the reaction efficiencies (mean ± SD, *N*=3).

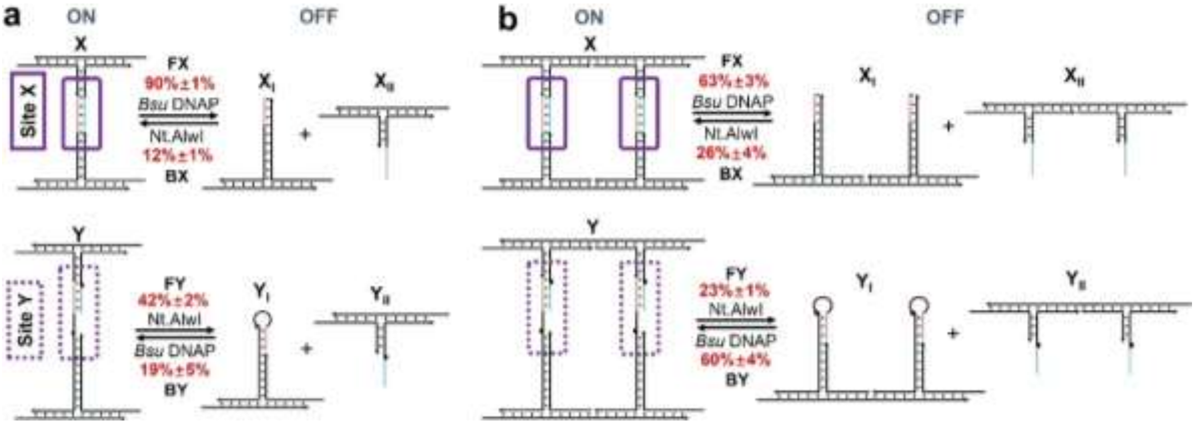

Supplementary Figure 12. Summary of minimalist system reaction yields. Switches X and Y in single-pair (a) and dual-pair (b) binding system. Working sites were located between two junctions outlined by purple squares (solid line: site X; dash line: site Y). Reaction efficiencies were shown in red on the corresponding reaction equations. Triplicated experiments were independently conducted (mean ± SD, N=3). The yield of FX for the dual-pair system dropped to 63% from 91% in the one-pair system. Similarly, the yield of FY for the dual-pair system dropped to 23% from 42% in the one-pair system. The yield drop is reasonable since the overall disassembly of the complexes required would not happen till two independent decoupling events happen at the same time. On the contrary, a yield increase of the backward reaction was presented in BX and BY reactions with two working sites, 26% (dual-pair) vs 12% (one-pair) for BX and 60% (dual-pair) vs 19% (one-pair) for BY. Presumably, two neighboring sticky ends of the same construct created in the backward reactions (BX or BY) enabled the assembly in a coordinated manner, in which the initial cohesion facilitated the subsequent one due to a kinetic favor. Prolonged incubation time led to an improved efficiency of the Nt. AlwI reactions, but resulted in more aggregates for *Bsu* DNAP reactions (results not shown). Therefore, we set the incubation time as 24 h for Nt. AlwI reactions and 2 h for *Bsu* DNAP reactions.

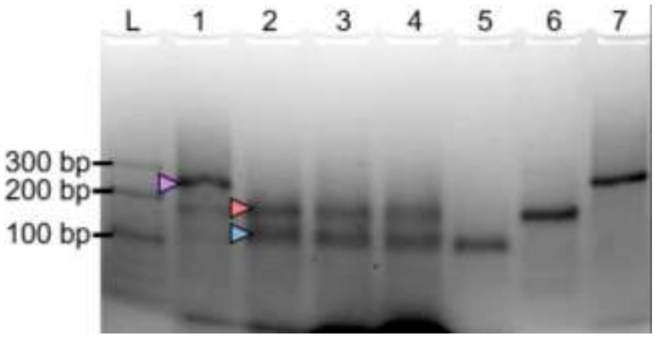

Supplementary Figure 13. Native polyacrylamide gel electrophoresis results of the reversible switch of type X in the dual-pair binding system. Lane L: 20 bp ladder. Lane 1: BX reaction. Structural partners X_I and X_{II} were fused into complex X with Nt.AlwI. Lane 2: FX reaction. Product X of Lane 1 was separated into partners X_I and X_{II} again by *Bsu* DNAP. Then the decoupled partners were further mixed with Nt.AlwI once again for 1 h (Lane 3) and 4 h (Lane 4), but no recoupled products were observed. Lane 5: X_{II}. Lane 6: X_I. Lane 7: X.

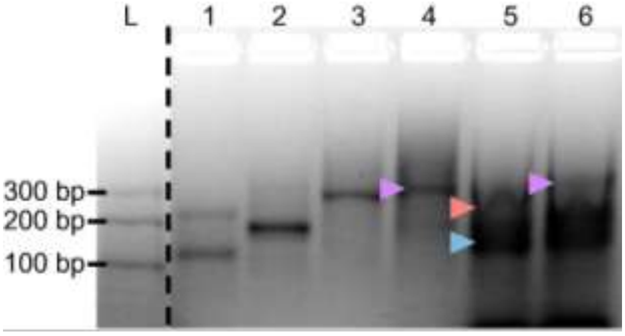

Supplementary Figure 14. Native polyacrylamide gel electrophoresis results of the reversible switch of type Y in the dual-pair binding system. Lane L: 20 bp ladder. Lane 1: Y_I. Lane 2: Y_{II}. Lane 3: Y. Lane 4: BY reaction. Structural partners Y_I and Y_{II} were fused into complex Y with *Bsu* DNAP. Lane 5: FY reaction. Product Y of Lane 4 was separated into partners Y_I and Y_{II} again by Nt.AlwI. Then the decoupled partners were further mixed with *Bsu* DNAP once again for 1 h (Lane 6), and they fused back into complete Y with a shallow band.

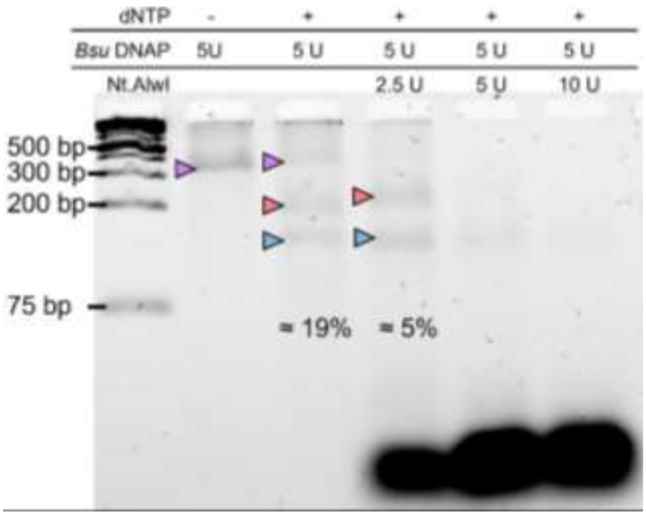

Supplementary Figure 15. Native polyacrylamide gel electrophoresis results of competition between *Bsu* DNAP and Nt.AlwI in the dual-pair binding system. 5-unit (U) *Bsu* DNAP was incubated in complex X without/with dNTP, and with 2.5-unit, 5-unit, and 10-unit Nt.AlwI from left to right. Once the Nt.AlwI was added the structures worked as templates to produce short strands (at the bottom of the gel) caused by the combined effect of two enzymes, like strand displacement amplification. Thus, the disassembly of structures was discouraged when both enzymes were supplied on purpose and by-products occurred. The reaction yields (numbers in the gel) dramatically dropped to ≈ 5% with a 2:1 ratio of *Bsu* DNAP to Nt.AlwI addition. Marker: 1 kb+ ladder.

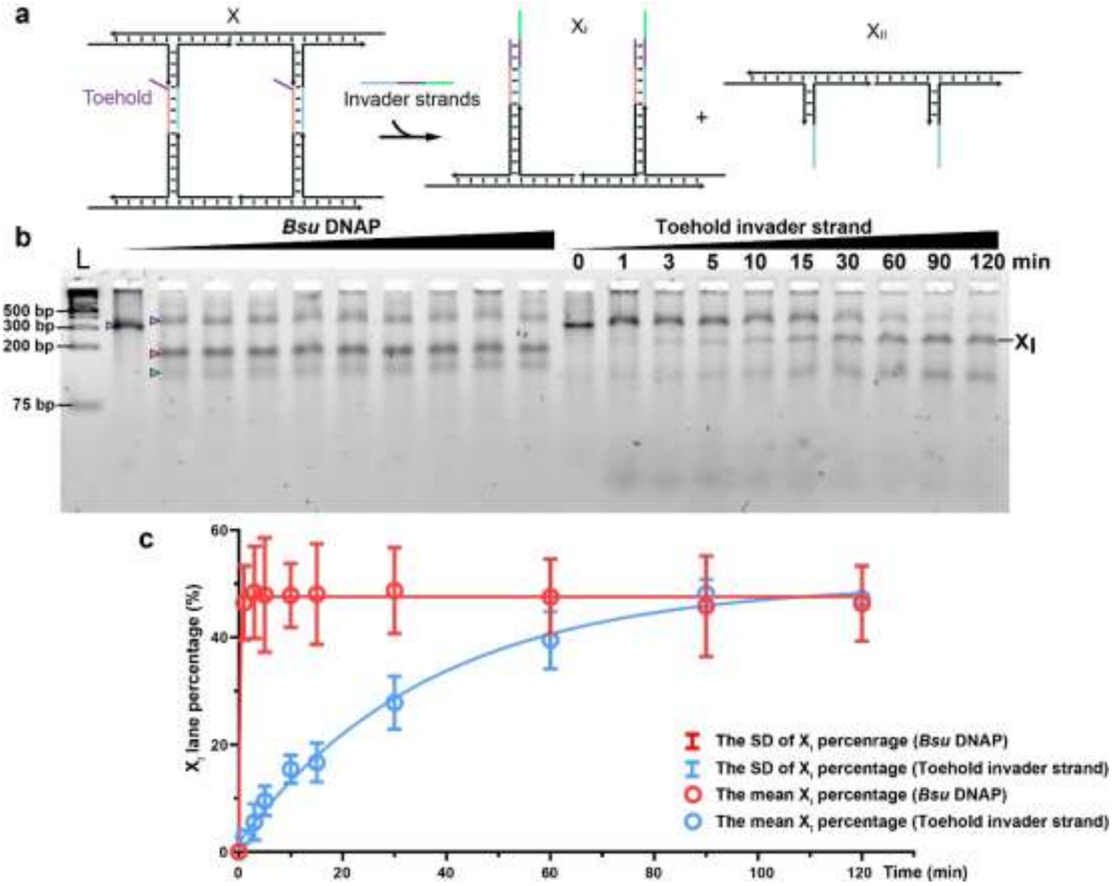

Supplementary 16. Comparison of the enzyme-mediated system and toehold-mediated strand displacement system in the FX reaction of the dual-pair binding system. In the enzyme-mediated reaction, the complex X, at a concentration of 250 nM, was mixed with 5 U *Bsu* DNAP for incubation at 37 °C for 2 s. In a toehold-mediated reaction (a), the complex X with the toehold, also at a concentration of 250 nM, was mixed with 250 nM toehold invader strands for incubation at 37 °C for 2 h. (b) Native polyacrylamide gel electrophoresis results of the time course for *Bsu* DNAP enzyme and toehold invader strand. L: 1 kb+ ladder. (c) Time-dependent FX reaction in *Bsu* DNAP enzyme (red dots) and toehold invader strand displacement (blue dots), resolved by native polyacrylamide gel electrophoresis. The percentage of X_I was quantified by an exponential fit. The reaction rate constants are 3 min^{-1} (± 0.65) and 0.03 min^{-1} (± 0.004) for enzyme- and toehold-mediated systems respectively, extracted from curves. Three independent experiments of each time point in both enzyme- and toehold-mediated were conducted (mean ± SD, N=3).

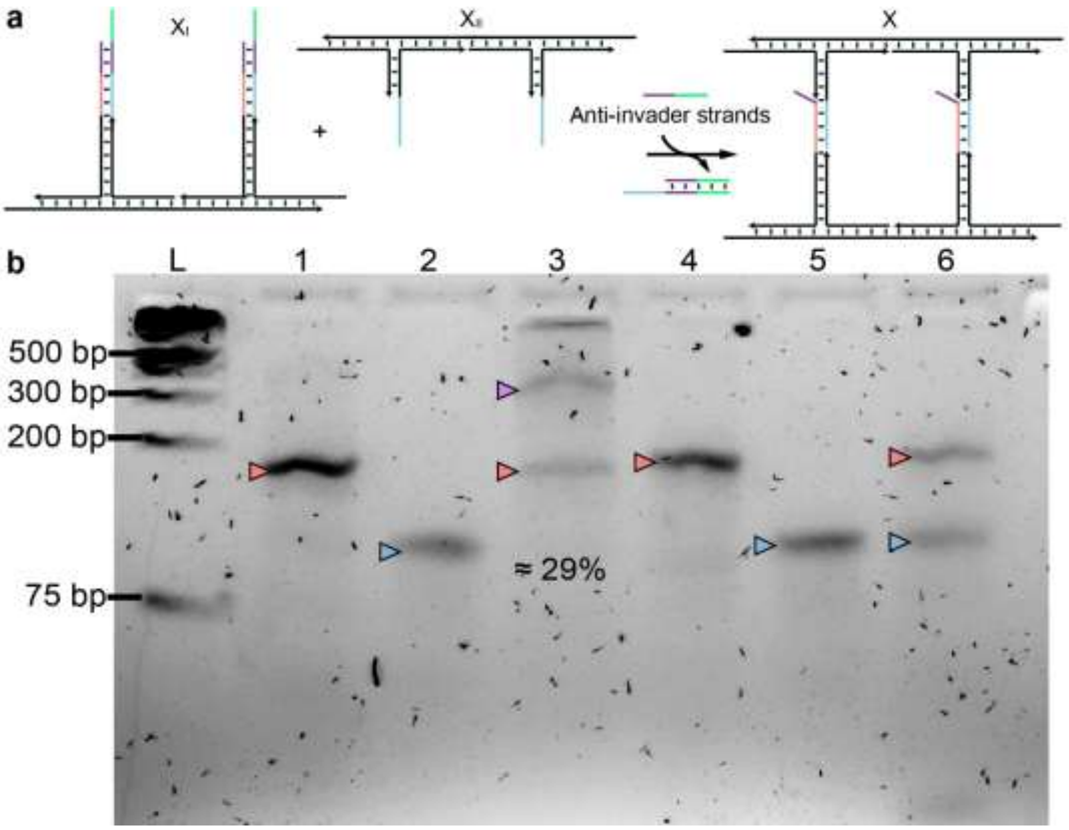

Supplementary Figure 17. Comparison of the enzyme-mediated system and toehold-mediated strand displacement system in the BX reaction of the dual-pair binding system. In the enzyme-mediated reaction, the X_I and X_{II}, at a concentration of 750 nM, were mixed with 15 U Nt.AlwI enzyme for an incubation at 37 °C overnight. In a toehold-mediated reaction (a), the X_I and X_{II} with toehold, also at a concentration of 750 nM, were mixed with 750 nM toehold invader strands for incubation at 37 °C overnight. (b) Native polyacrylamide gel electrophoresis results of the dual-pair binding system BX reaction. Lane L: 1 kb+ ladder. Lane 1: dual-pair binding site unit X_I. Lane 2: dual-pair binding site unit X_{II}. Lane 3: the formation of complex X (indicated by the purple triangle, yield ≈ 29%) from X_I and X_{II} upon Nt.AlwI supplied. Lane 4: dual-pair binding site unit X_I with toehold. Lane 5: dual-pair binding site unit X_{II} with toehold. Lane 6: the formation failure of complex X from X_I and X_{II} with toehold upon anti-invader strands supplied.

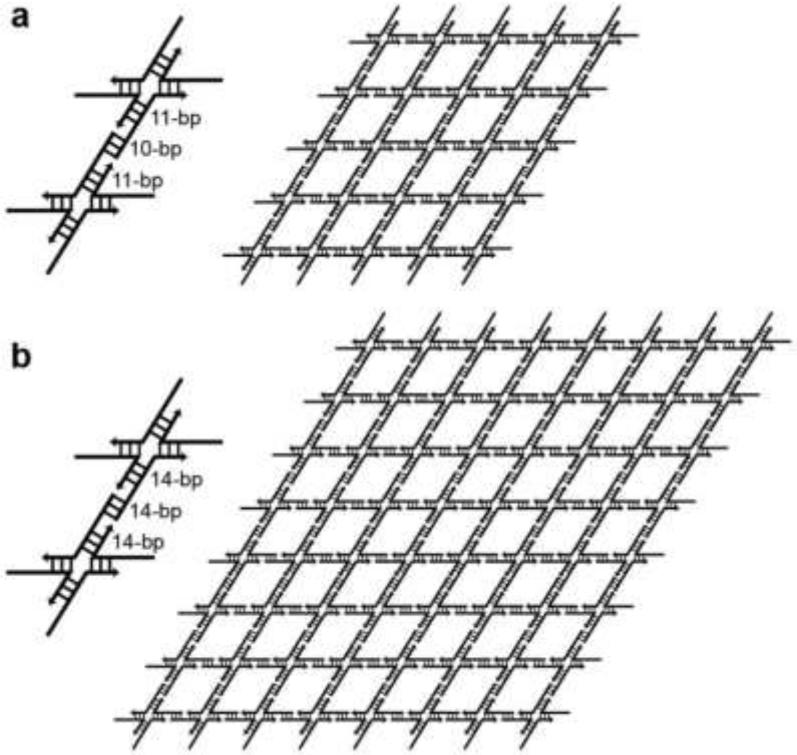

Supplementary Figure 18. Schematics of 4-arm junction lattice. (a) 5×5 lattice. The length of the 4-arm junction is 32-bp per strand. (b) 8×8 lattice. The length of the 4-arm junction is 42-bp per strand.

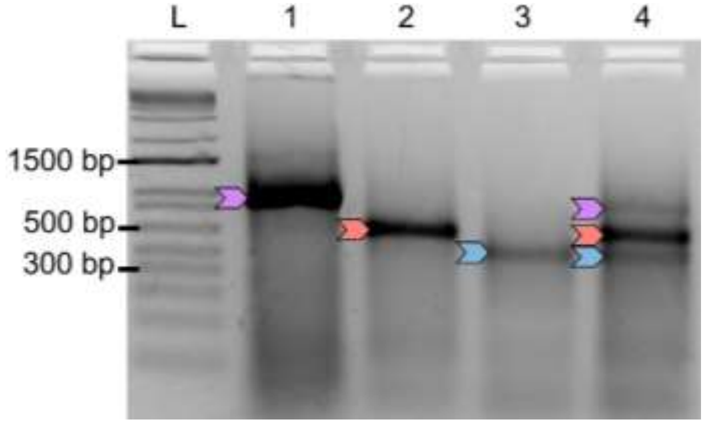

Supplementary Figure 19. Native polyacrylamide gel electrophoresis results of the 5×5 lattice, 3×5 lattice and 2×5 lattice. Lane L: 1 kb+ ladder. Lane 1: 5×5 lattice. Lane 2: 3×5 lattice. Lane 3: 2×5 lattice. Lane 4: the mixture of three types of lattices. The arrows with black edges in red, blue and purple indicate the product bands, respectively.

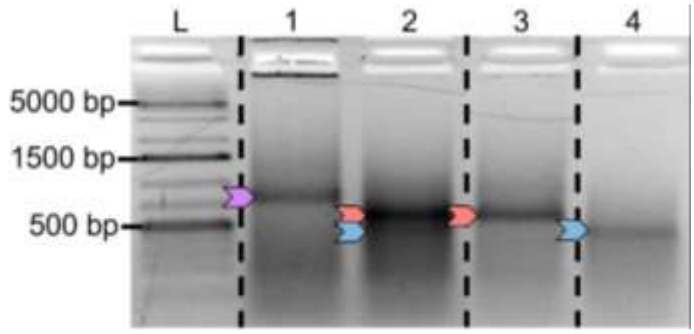

Supplementary Figure 20. Native polyacrylamide gel electrophoresis results of FX reaction in the 5×5 lattice. Lane L: 1 kb+ ladder. Lane 1: 5×5 lattice X. Lane 2: FX reaction with yield 32% (± 0.2%) (Profile Min. as background). 5×5 lattice (complex X) was added with *Bsu* DNAP at 37 °C for 4 h and divided into 3×5 lattice X_I. and 2×5 lattice X_{II}. Lane 3: 3×5 lattice X_I. Lane 4: 2×5 lattice X_{II}. The arrows with black edges in red, blue and purple indicate the product bands of X_I, X_{II} and X respectively, which apply to the rest of Supplementary unless otherwise stated. Triplicated experiments were independently conducted (mean ± SD, *N*=3).

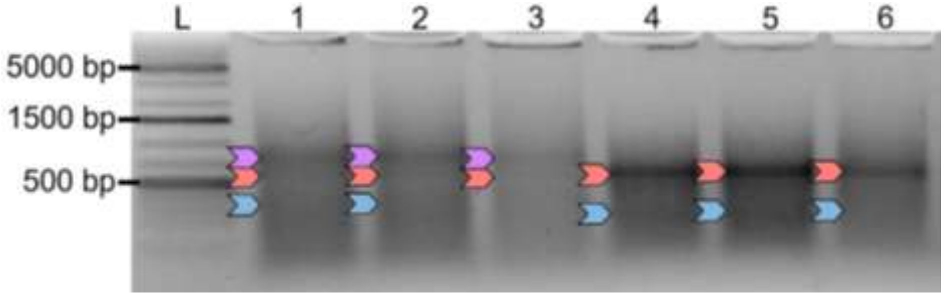

Supplementary Figure 21. Native polyacrylamide gel electrophoresis results of BX reaction in the 5×5 lattice. Lane L: 1 kb+ ladder. Lanes 1, 2 and 3: BX reaction with yield 16% (± 2%) (Profile Min. as background). The separated lattices X_I and X_{II} were assembled with Nt.AlwI under 37 °C 38.5 °C and 40 °C, respectively. The partners were fused into a complete complex X at different temperatures. Lanes 4, 5 and 6: the corresponding controls without the enzyme under the same corresponding temperatures. Triplicated experiments were independently conducted (mean ± SD, *N*=3).

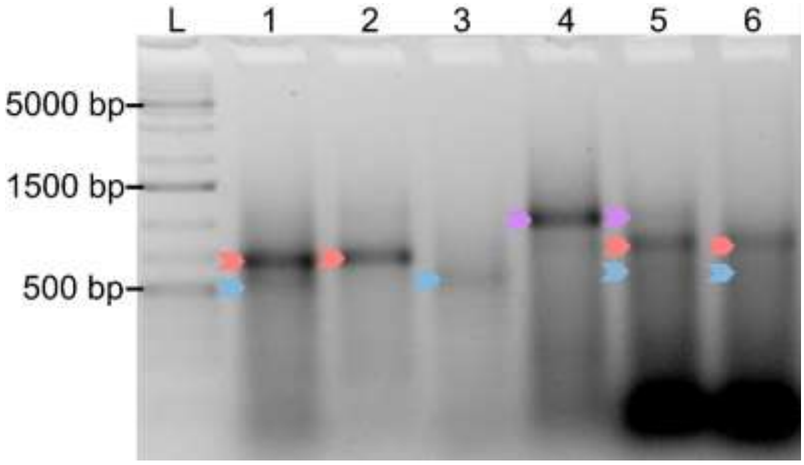

Supplementary Figure 22. Native polyacrylamide gel electrophoresis results of FY reaction in the 5×5 lattice. Lane L: 1 kb+ ladder. Lane 1: 3×5 lattice Y_I was mixed with 2×5 lattice Y_{II} without enzyme. Lane 2: 3×5 lattice Y_I. Lane 3: 2×5 lattice Y_{II}. Lane 4: 5×5 lattice Y. Lanes 5 and 6: the product of FY reaction (with 2 µl and 4 µl Nt.AlwI, respectively). The complex Y was divided into two lattices. The yield of FY is 17% (± 0.5%) (Profile Min. as background). The arrows in red, blue and purple indicate the product bands of Y_I, Y_{II} and Y respectively, which apply to the rest Supplementary unless otherwise stated. Triplicated experiments were independently conducted (mean ± SD, *N*=3).

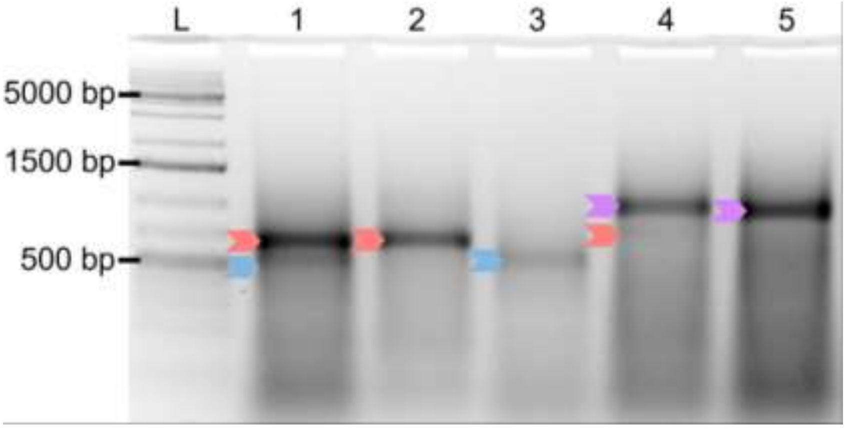

Supplementary Figure 23. Native polyacrylamide gel electrophoresis results of BY reaction in the 5×5 lattice. Lane L: 1 kb+ ladder. Lane 1: 3×5 lattice Y_I was mixed with 2×5 lattice Y_{II} without enzyme. Lane 2: 3×5 lattice Y_I. Lane 3: 2×5 lattice Y_{II}. Lane 4: the product of BY reaction with yield 18% (± 0.2%) (Profile Min. as background). Separate partners Y_I and Y_{II} were mixed with *Bsu* DNAP to fuse to complete Y showed in a main band in the lane. Lane 5: 5×5 lattice Y. Triplicated experiments were independently conducted (mean ± SD, *N*=3).

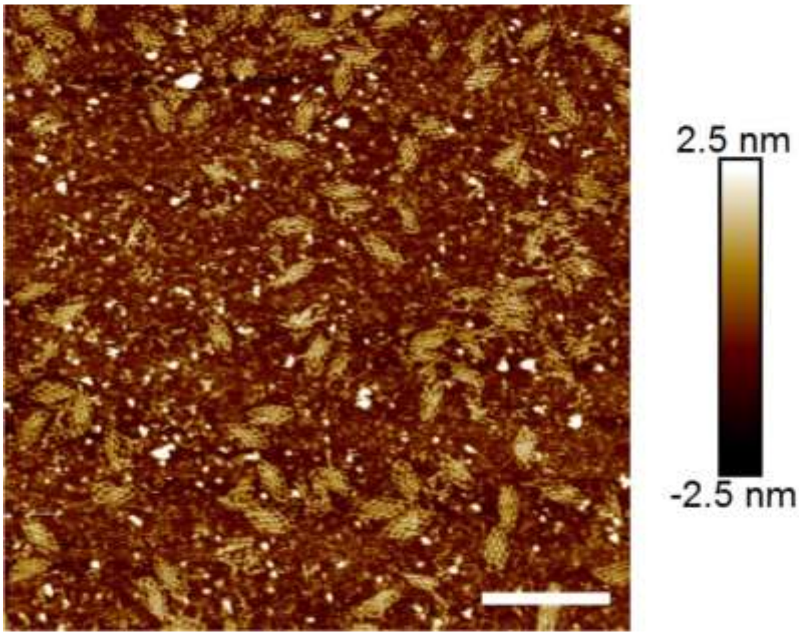

Supplementary Figure 24. AFM result of 5×5 lattice complex X. Scale bar: 200 nm.

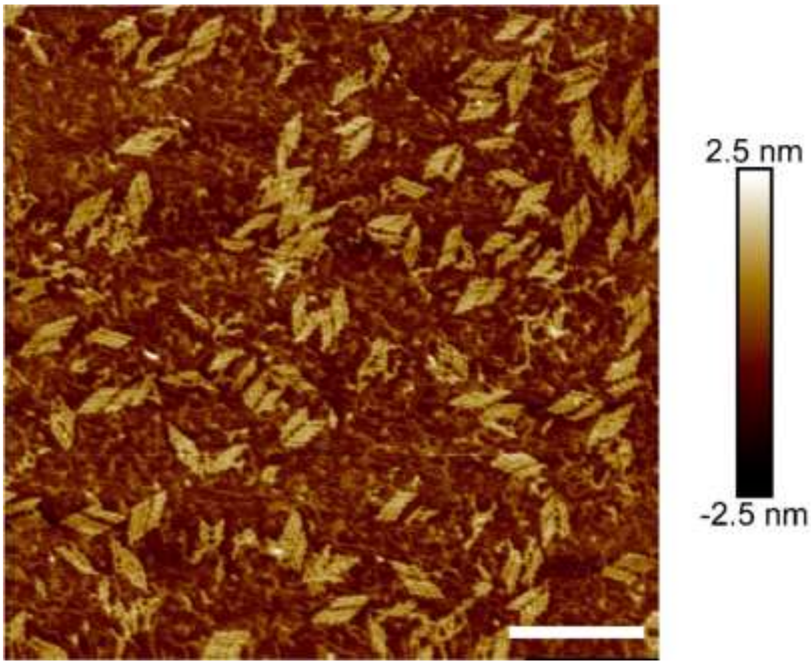

Supplementary Figure 25. AFM result of 5×5 lattice complex Y. The working sites between columns two and three were longer than the regular arms. The AFM imaging of Y was different from the complex of X in Supplementary Fig. 24. Scale bar: 200 nm.

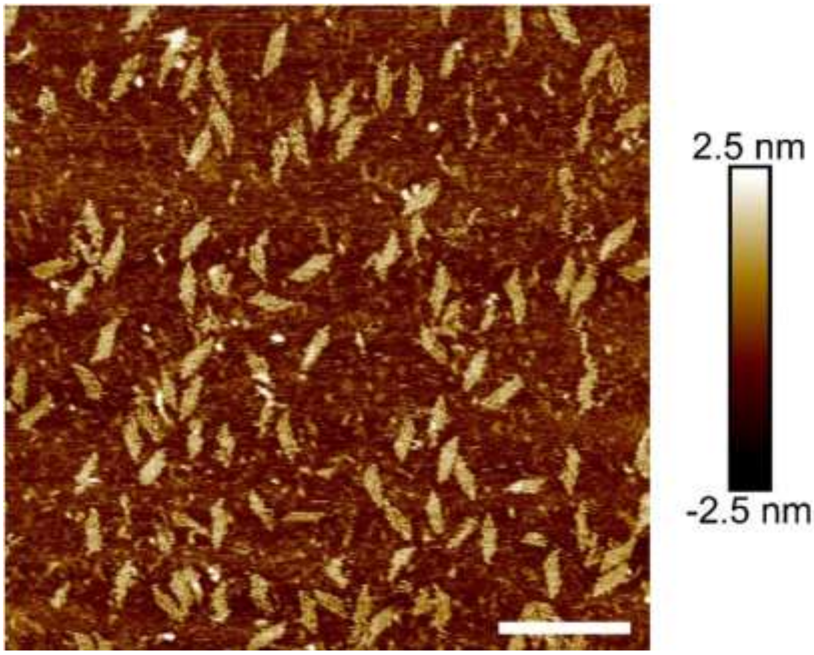

Supplementary Figure 26. AFM result of 3×5 lattice. Scale bar: 200 nm.

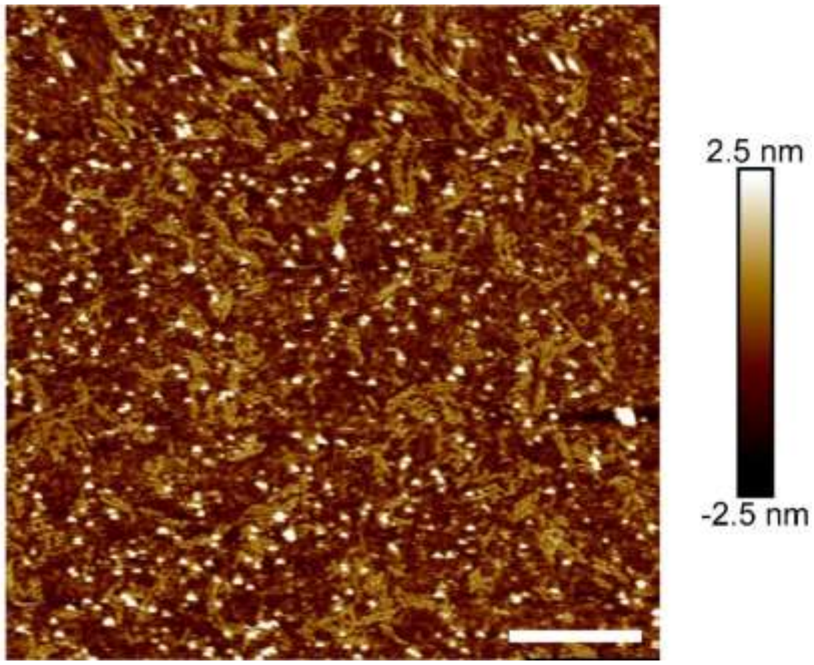

Supplementary Figure 27. AFM result of 2×5 lattice. Scale bar: 200 nm.

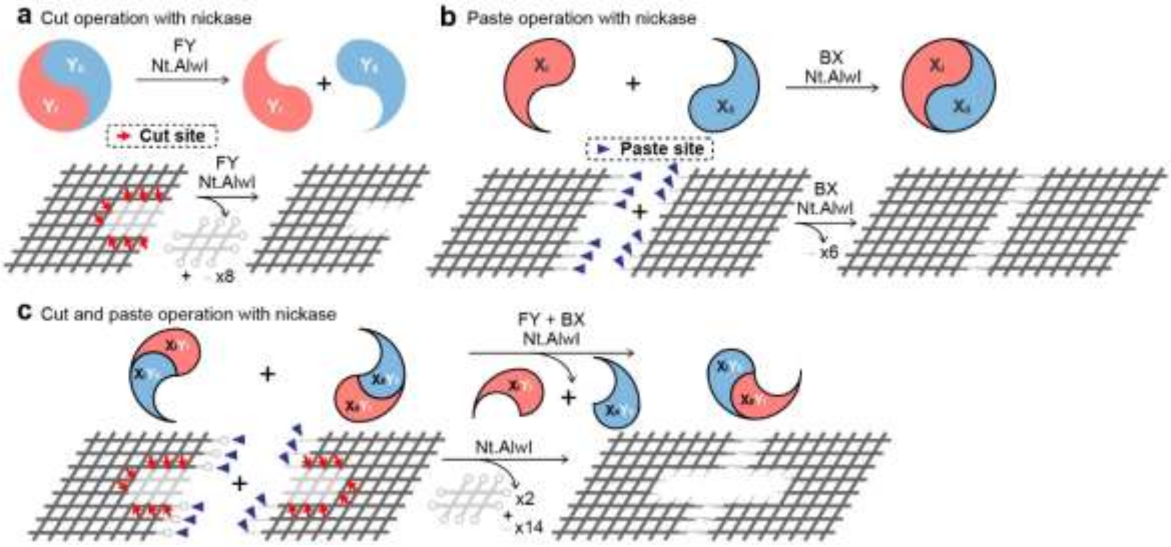

Supplementary Figure 28. Schematics one-step cut and paste on 8×8 lattice by Nt.AlwI nicking endonuclease. (a) Schematic of cut operation with nicking endonuclease (e.g., FY reaction) to create a C-shape lattice. (b) Schematic of paste operation with nicking endonuclease (e.g., BX reaction) to form an 8×16 lattice. (c) Schematic of the composite cut (FY reaction) and paste (BX reaction) operation with nicking endonuclease to rearrange a 0-shaped lattice. The cropped lattices are different from the system with *Bsu* DNAP.

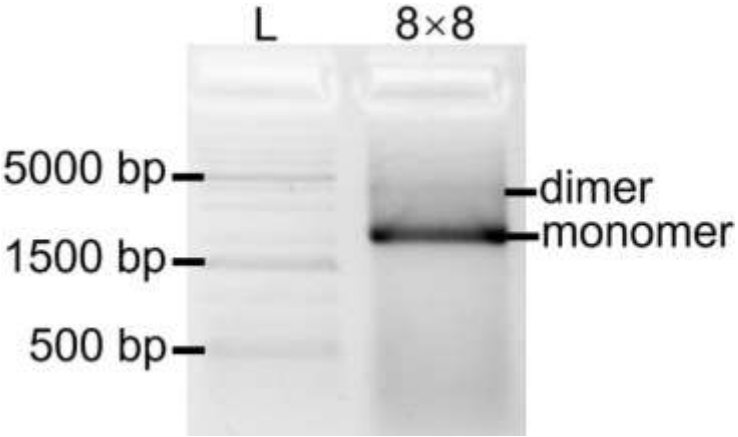

Supplementary Figure 29. Native polyacrylamide gel electrophoresis result of 8×8 lattice. Lane L: 1 kb+ ladder. Lane 8×8: complete 8×8 lattice.

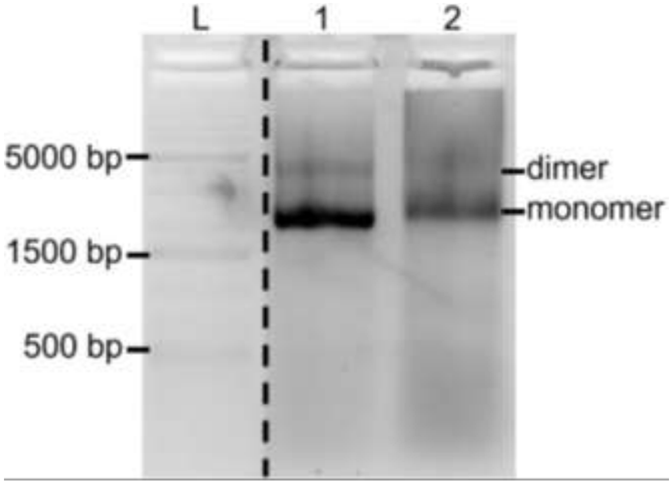

Supplementary Figure 30. Native polyacrylamide gel electrophoresis results of C-shaped lattice. Lane L: 1 kb+ ladder. Lane 1: 8×8 lattice. Lane 2: cut of C-shaped lattice. The 8×8 lattice was designed with 8 cut sites and mixed with *Bsu* DNAP. Due to the shape of the C-shaped monomer being different, the band of the monomer was shifted slightly higher than that of the original 8×8 lattice.

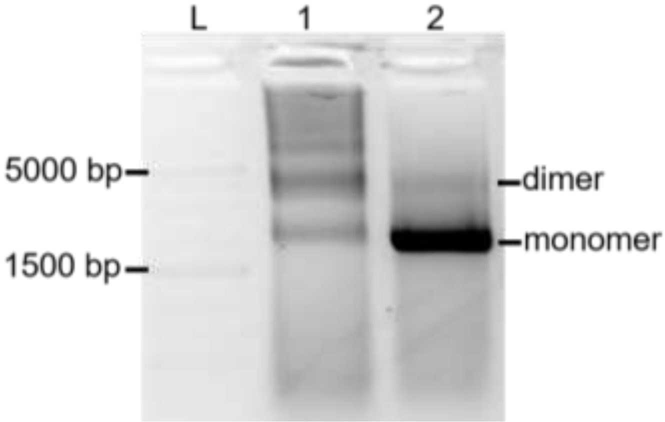

Supplementary Figure 31. Native polyacrylamide gel electrophoresis results of two 8×8 lattices coupled. Lane L: 1 kb+ ladder. Lane 1: 8×16 lattices. Two 8×8 lattices were designed with 6 pairs of paste sites at the edge and mixed with *Bsu* DNAP to paste together. Exposure of vast overhang strands in the 8×8 lattice might cause the undesired polymers in the lane. Lane 2: 8×8 lattice.

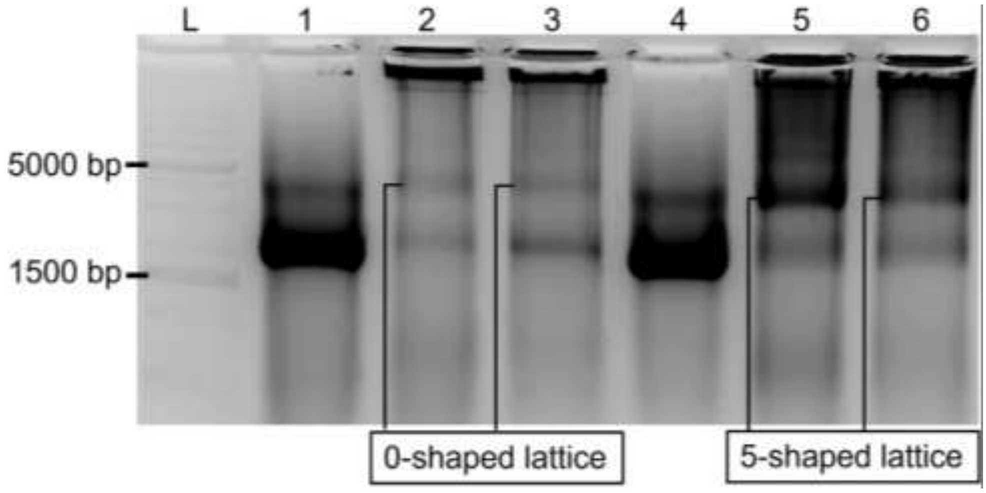

Supplementary Figure 32. Native polyacrylamide gel electrophoresis results of the 0- and 5-shaped lattices. Lane L: 1 kb+ ladder. Lane 1: 0-shaped lattice control. Two 8×8 lattices were designed to convert into a 0-shaped lattice with *Bsu* DNAP but no dTTP/dGTP/dCTP. Lanes 2 and 3: 0-shaped lattice. Two 8×8 lattices were designed to convert into a 0-shaped lattice with *Bsu* DNAP and dTTP/dGTP/dCTP. Lane 4: 5-shaped lattice control. Lanes 5 and 6: 5-shaped lattice.

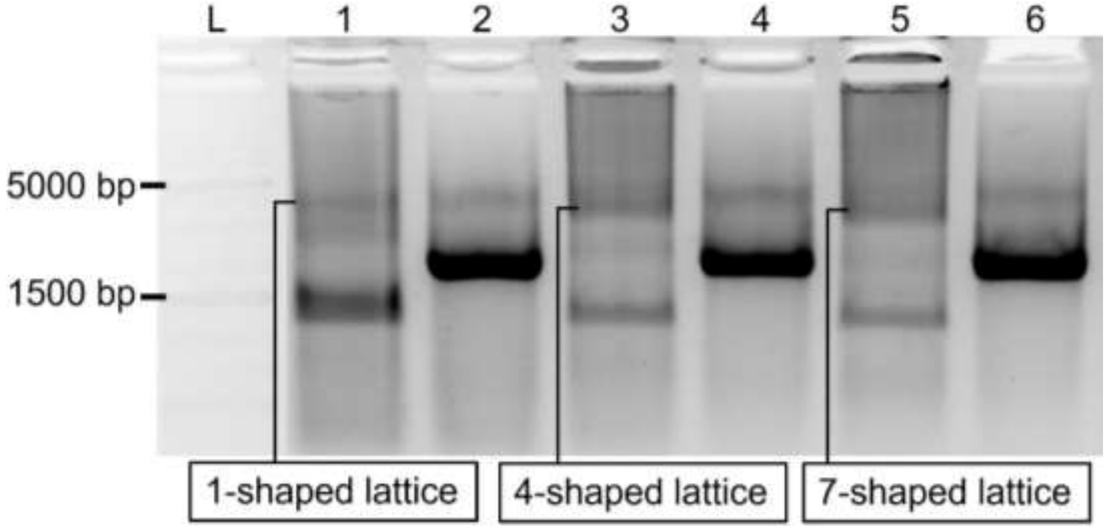

Supplementary Figure 33. Native polyacrylamide gel electrophoresis results of the 1-, 4- and 7-shaped lattices. Lane L: 1 kb+ ladder. Lane 1:1-shaped lattice. Two 8×8 lattices were designed to convert into a 1-shaped lattice with *Bsu* DNAP and dTTP/dGTP/dCTP. Lane 2: 1-shaped lattice control. Two 8×8 lattices were designed to convert into a 1-shaped lattice with *Bsu* DNAP but no dTTP/dGTP/dCTP. Lane 3: 4-shaped lattice. Lane 4: 4-shaped lattice control. Lane 5: 7-shaped lattice. Lane 6: 7-shaped lattice control.

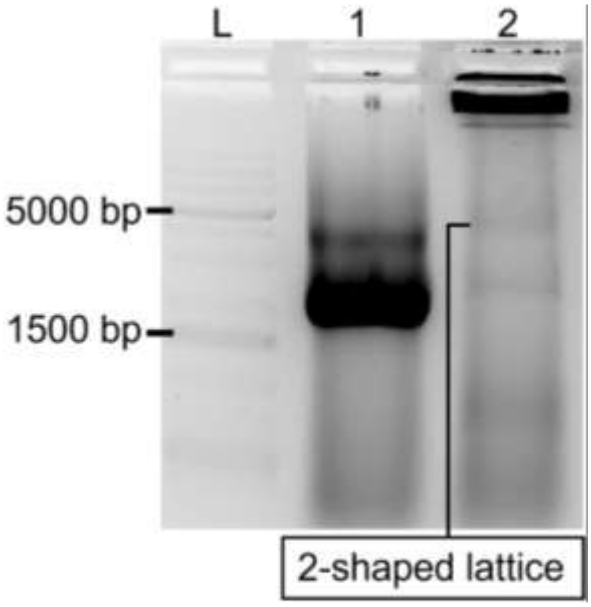

Supplementary Figure 34. Native polyacrylamide gel electrophoresis results of the 2-shaped lattice. Lane L: 1 kb+ ladder. Lane 1: 2-shaped lattice control. Two 8×8 lattices were designed to convert into a 2-shaped lattice with *Bsu* DNAP but no dTTP/dGTP/dCTP. Lane 2: 2-shaped lattice. Two 8×8 lattices were designed to convert into a 2-shaped lattice with *Bsu* DNAP and dTTP/dGTP/dCTP.

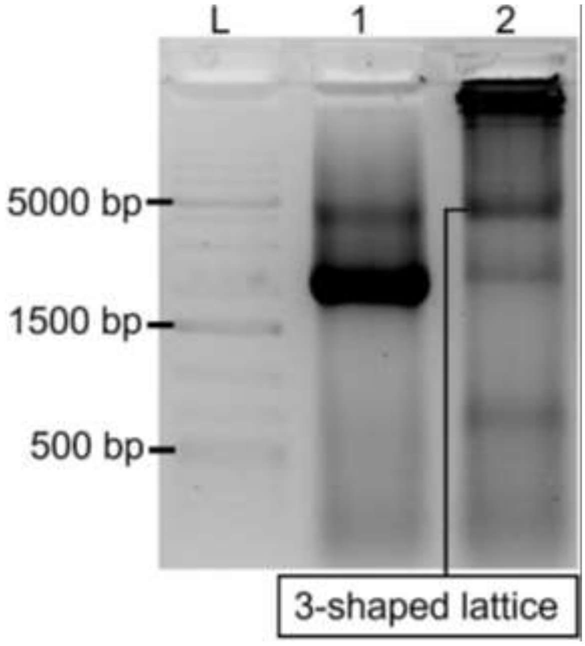

Supplementary Figure 35. Native polyacrylamide gel electrophoresis results of the 3-shaped lattice. Lane L: 1 kb+ ladder. Lane 1: 3-shaped lattice control. Two 8×8 lattices were designed to convert into a 3-shaped lattice with *Bsu* DNAP but no dTTP/dGTP/dCTP. lane 2: 3-shaped lattice. Two 8×8 lattices were designed to convert into a 3-shaped lattice with *Bsu* DNAP and dTTP/dGTP/dCTP.

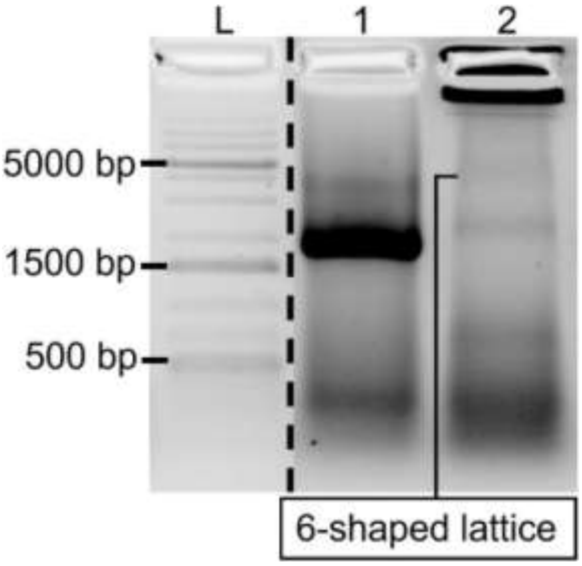

Supplementary Figure 36. Native polyacrylamide gel electrophoresis results of the 6-shaped lattice. Lane L: 1 kb+ ladder. Lane 1: 6-shaped lattice control. Two 8×8 lattices were designed to convert into a 6-shaped lattice with *Bsu* DNAP but no dTTP/dGTP/dCTP. lane 2: 6-shaped lattice. Two 8×8 lattices were designed to convert into a 6-shaped lattice with *Bsu* DNAP and dTTP/dGTP/dCTP.

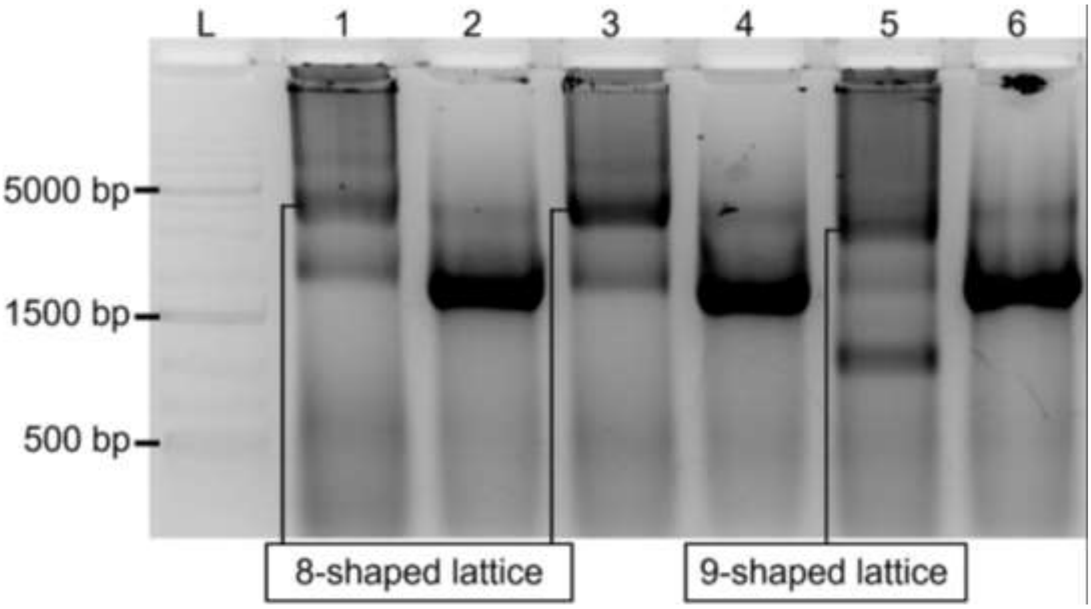

Supplementary Figure 37. Native polyacrylamide gel electrophoresis results of the 8- and 9-shaped lattices. Lane L: 1 kb+ ladder. Lane 1: 8-shaped lattice with six-pair working sites. Two 8×8 lattices were designed to convert into an 8-shaped lattice with *Bsu* DNAP and dTTP/dGTP/dCTP. Lanes 2 and 4: 8-shaped lattice control. Two 8×8 lattices were designed to convert into an 8-shaped lattice with *Bsu* DNAP but no dTTP/dGTP/dCTP. Lane 3: 8-shaped lattice with five-pair working sites. Lane 5: 9-shaped lattice. Lane 6: 9-shaped lattice control.

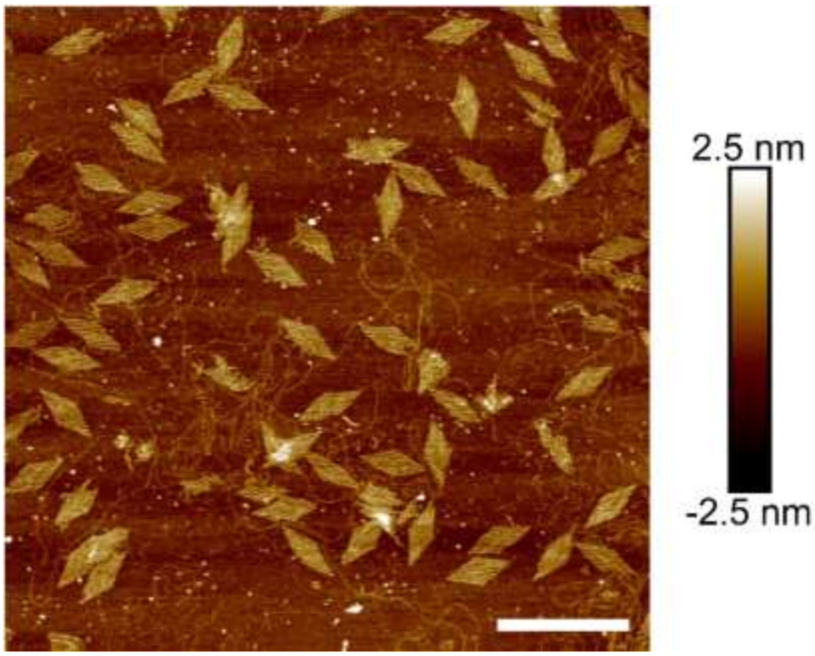

Supplementary Figure 38. AFM result of 8×8 lattice. Scale bar: 400 nm.

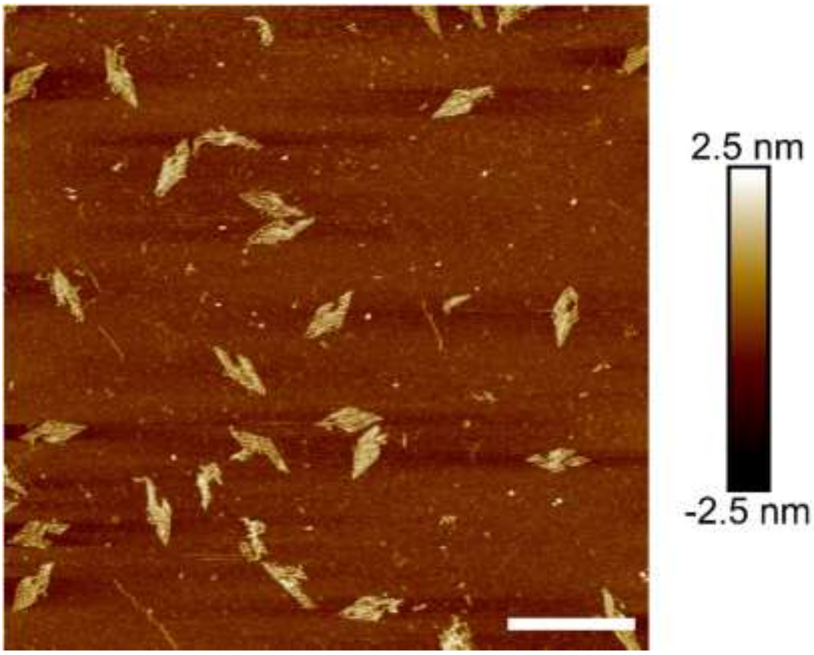

Supplementary Figure 39. AFM result of C-shaped lattice. Scale bar: 400 nm.

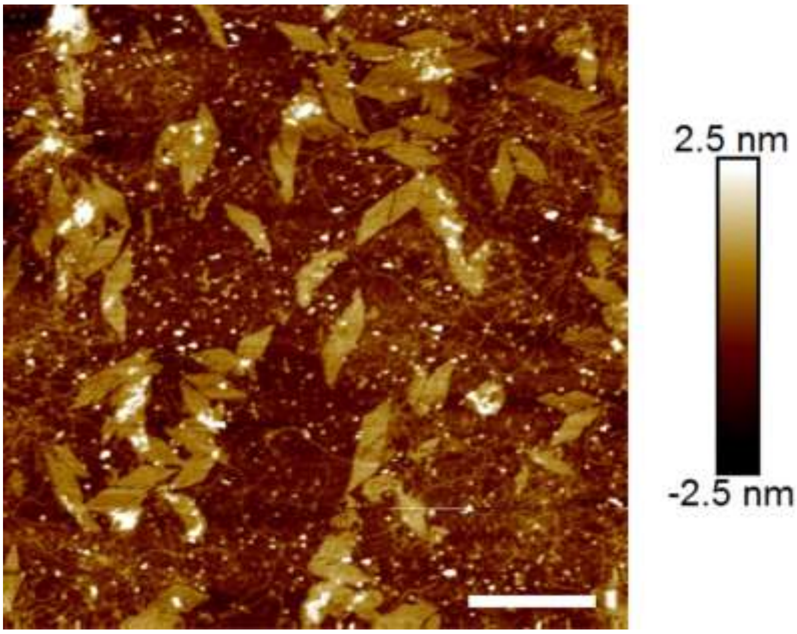

Supplementary Figure 40. AFM result of two 8×8 lattices coupled. Scale bar: 400 nm.

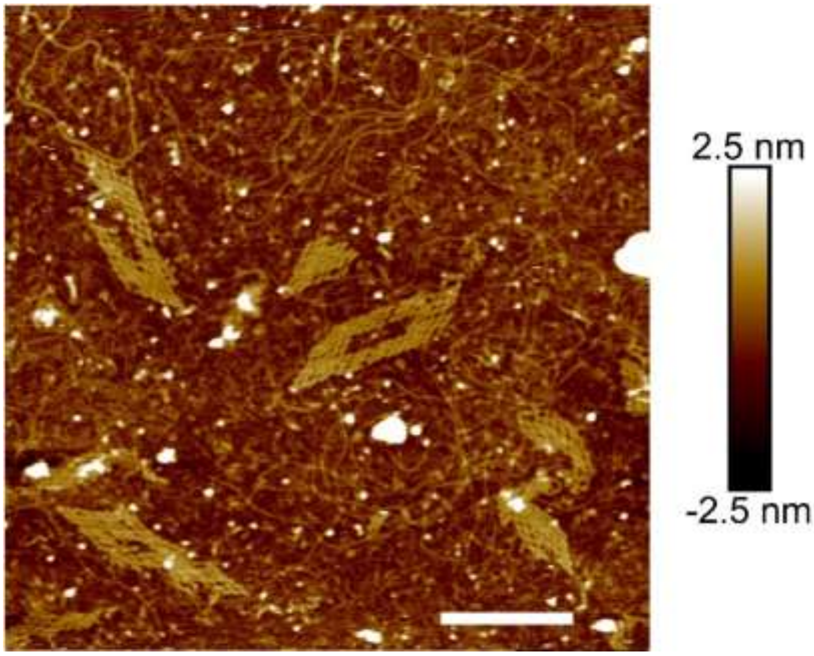

Supplementary Figure 41. AFM result of 0-shaped lattice. Scale bar: 200 nm.

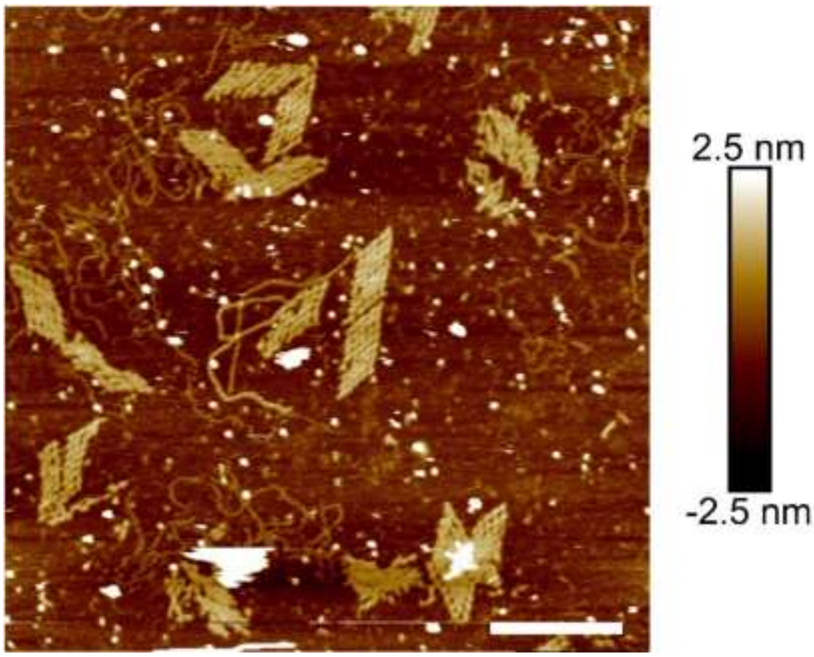

Supplementary Figure 42. AFM result of 1-shaped lattice. Scale bar: 200 nm.

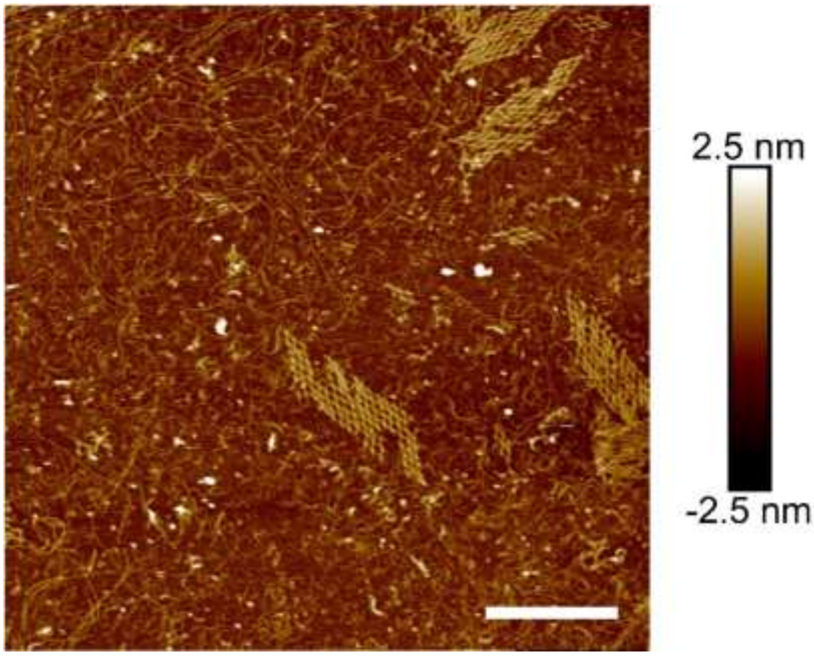

Supplementary Figure 43. AFM result of 2-shaped lattice. Scale bar: 200 nm.

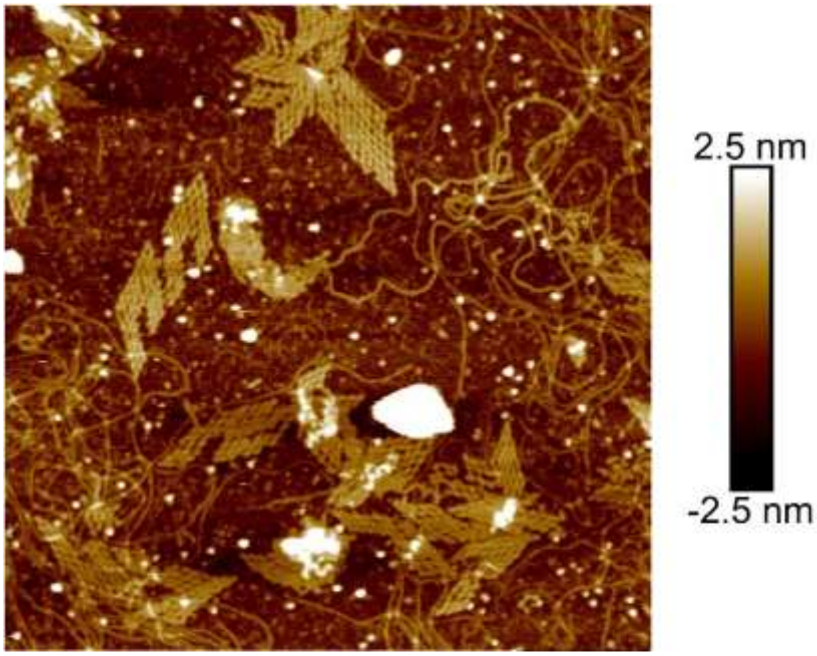

Supplementary Figure 44. AFM result of 3-shaped lattice. Scale bar: 200 nm.

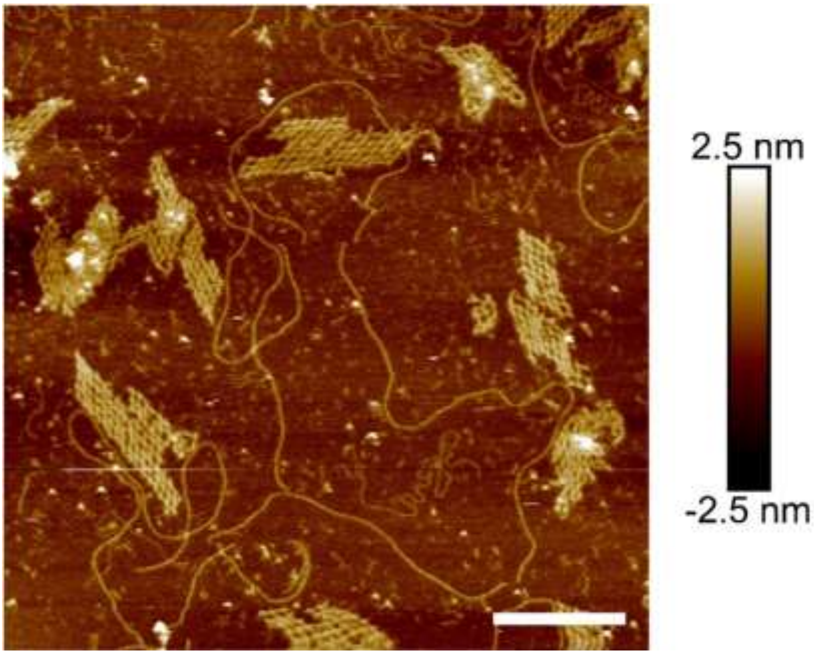

Supplementary Figure 45. AFM result of 4-shaped lattice. Scale bar: 200 nm.

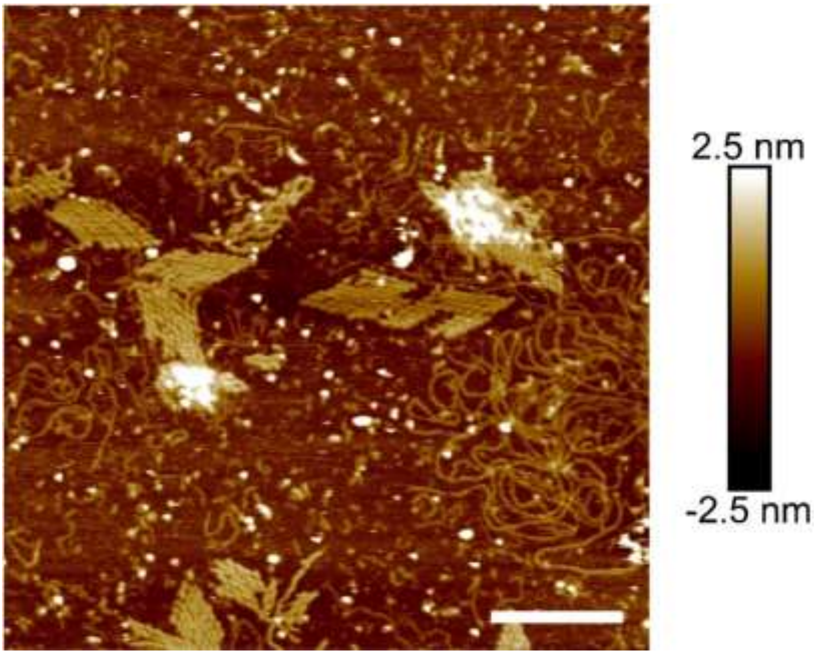

Supplementary Figure 46. AFM result of 5-shaped lattice. Scale bar: 200 nm.

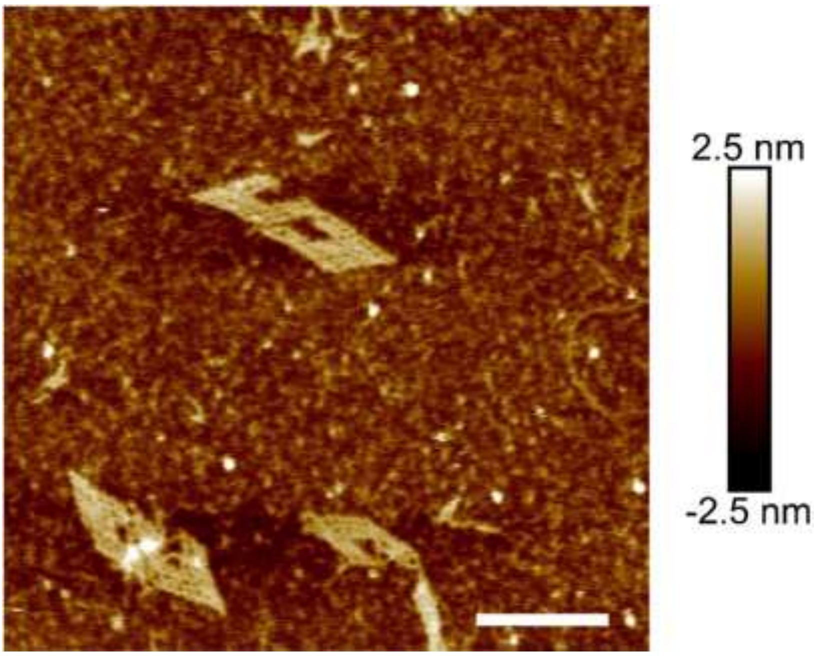

Supplementary Figure 47. AFM result of 6-shaped lattice. Scale bar: 200 nm.

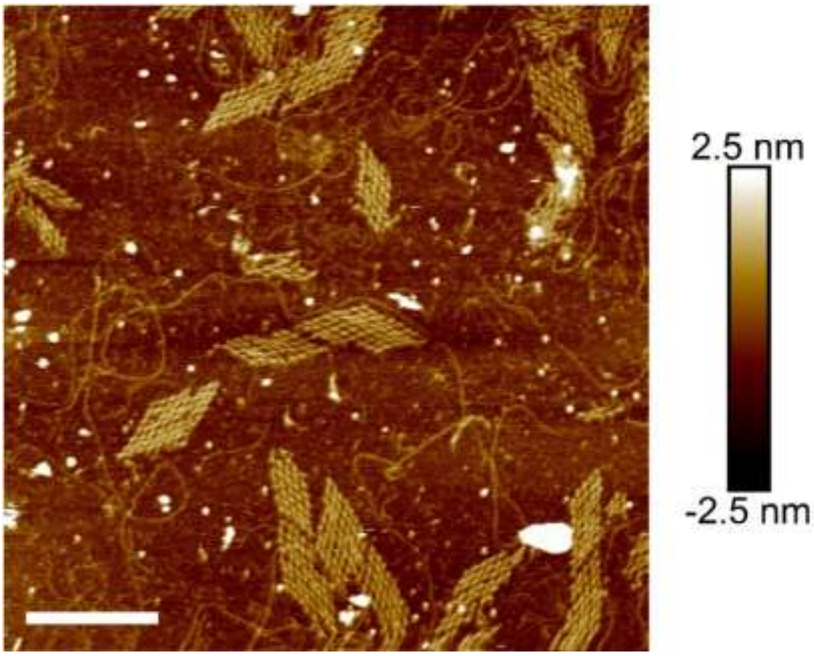

Supplementary Figure 48. AFM result of 7-shaped lattice. Scale bar: 200 nm.

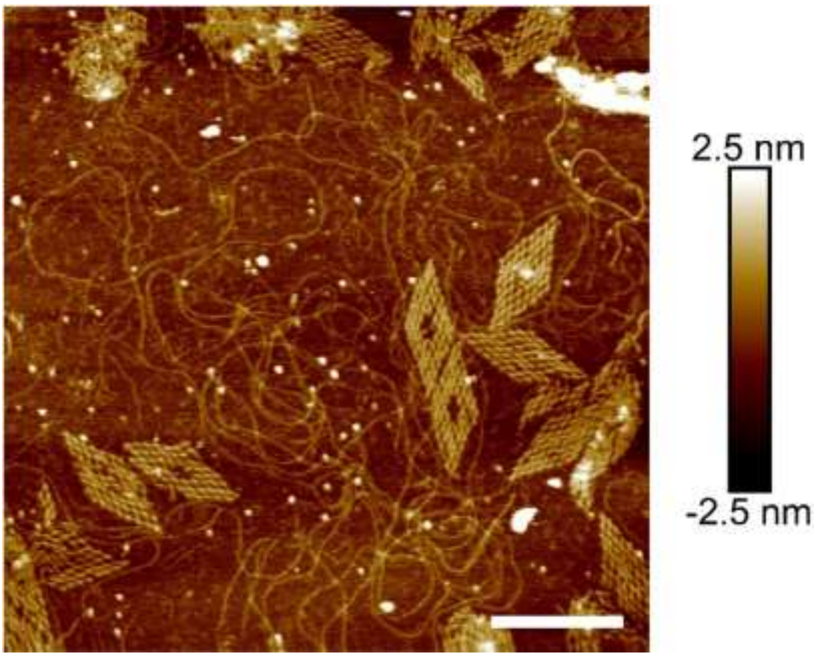

Supplementary Figure 49. AFM result of 8-shaped lattice. Scale bar: 200 nm.

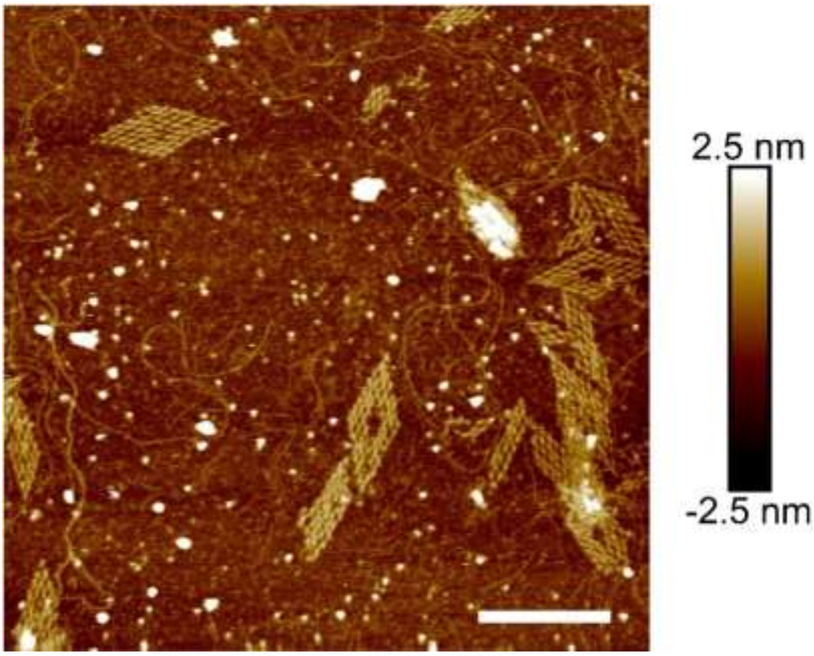

Supplementary Figure 50. AFM result of 9-shaped lattice. Scale bar: 200 nm.

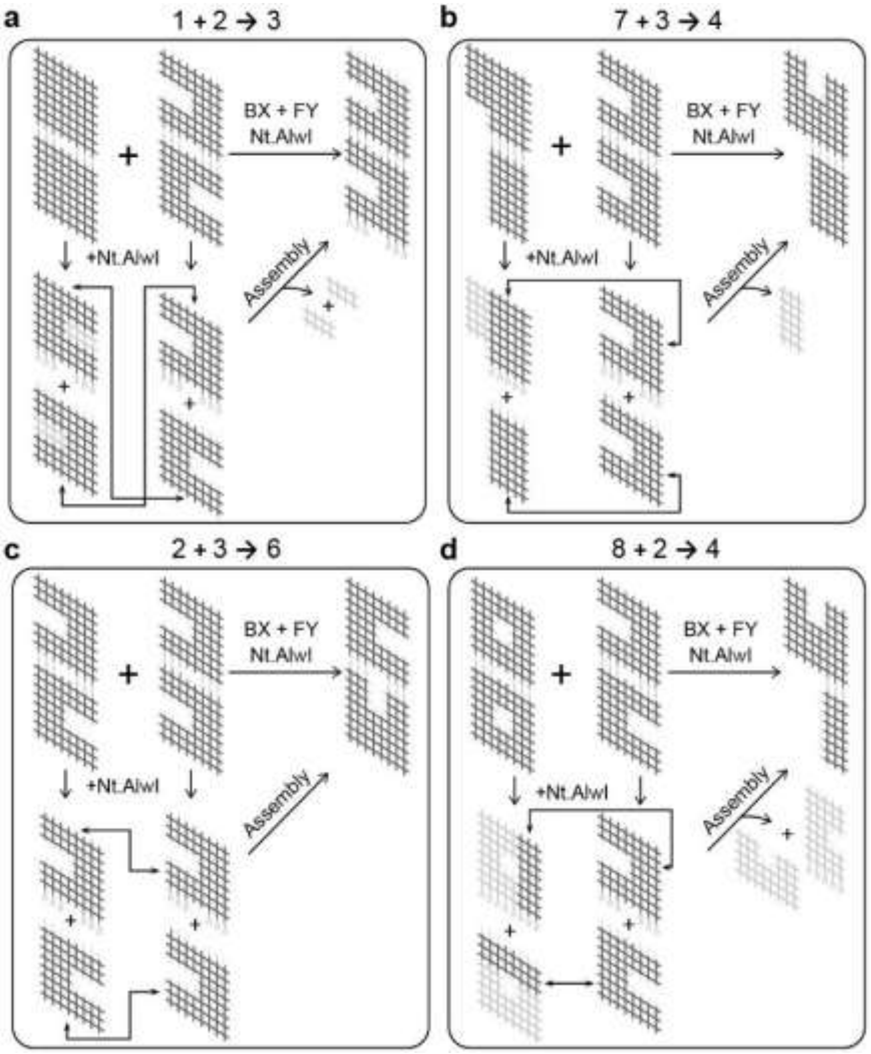

Supplementary Figure 51. Schematics of rearrangement of digit-shaped lattices by Nt.AlwI. The digit lattices could be mixed and rearranged into new digit lattices. The working sites of Nt.AlwI could be designed to realize the next round of one-step cut and paste operation (reactions BX and FY) as shown in Supplementary Fig. 28. (a) 1- and 2-digit lattices were decoupled into six sub-lattices and carved inside, then the edges bound to reform the 3-digit lattice. (b) Separate division of 7- and 3-digit lattices into five sub-lattices and the U- and I-shaped lattices can reform a 4-digit lattice. (c) 2- and 3-digit lattices could be split into different U-shapes with diverse edges, which could pair to form the new 6-digit lattice. (d) Also, an 8-digit lattice was mixed with a 2-digit lattice would be treated by Nt. AlwI into a 4-digit lattice.

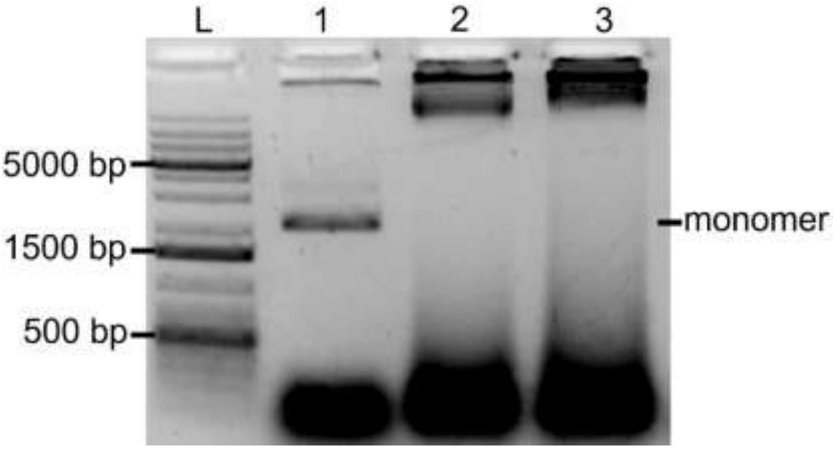

Supplementary Figure 52. Native polyacrylamide gel electrophoresis results of square origami without the protective TpT tails. Lane L: 1 kb+ ladder. Lane 1: the *Bsu* DNAP was added in the square origami without dNTPs. The origami structure was kept complete. Lanes 2 and 3: the *Bsu* DNAP was added to the square origami with 0.5 mM and 2 mM dNTPs, respectively. The square origami was destroyed and aggerated by the enzyme without the TpT tail protection.

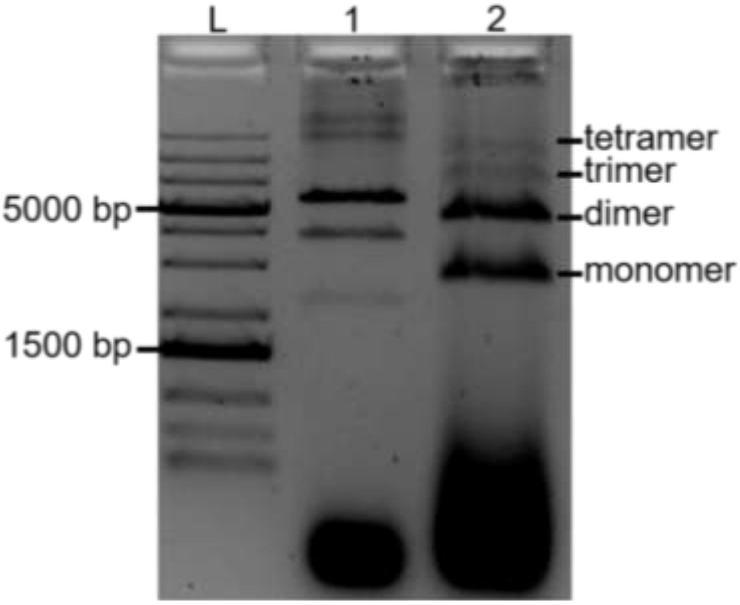

Supplementary Figure 53. Native polyacrylamide gel electrophoresis results of the L-shaped trimer converted from the I-shaped trimer. Lane L: 1 kb+ ladder. Lane 1: Before rearrangement, the origami units form an I-shaped trimer. Lane 2: After rearrangement, the I-shaped trimer was converted into an L-shaped trimer by *Bsu* DNAP with dTTP/dGTP/dCTP addition. With enzyme treatment, all bands slightly shift higher binding with protein, which is applied to the rest of Supplementary Figs of origami structures unless otherwise stated. The yield of the L-shaped trimer is only 3% (N=3). The origami units were mixed without gel purification, thus excess unformed staple strands existed at the bottom of the gel.

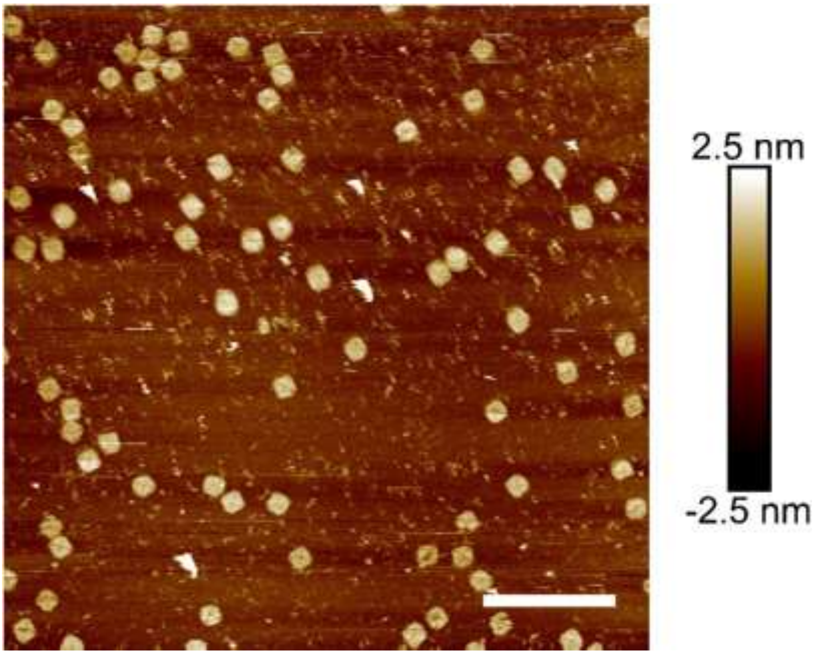

Supplementary Figure 54. AFM result of origami squares. Scale bar: 600 nm.

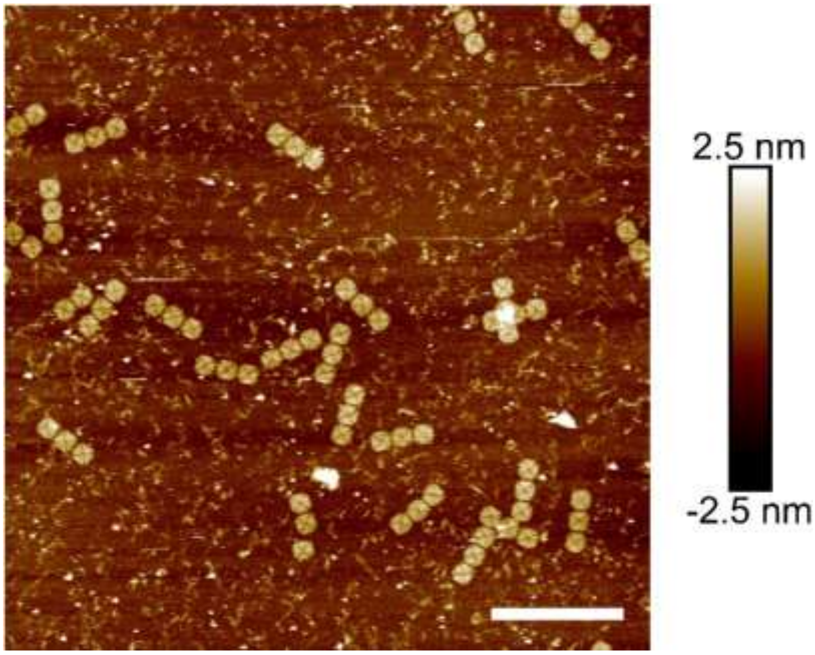

Supplementary Figure 55. AFM result of I-shaped trimer. Scale bar: 600 nm.

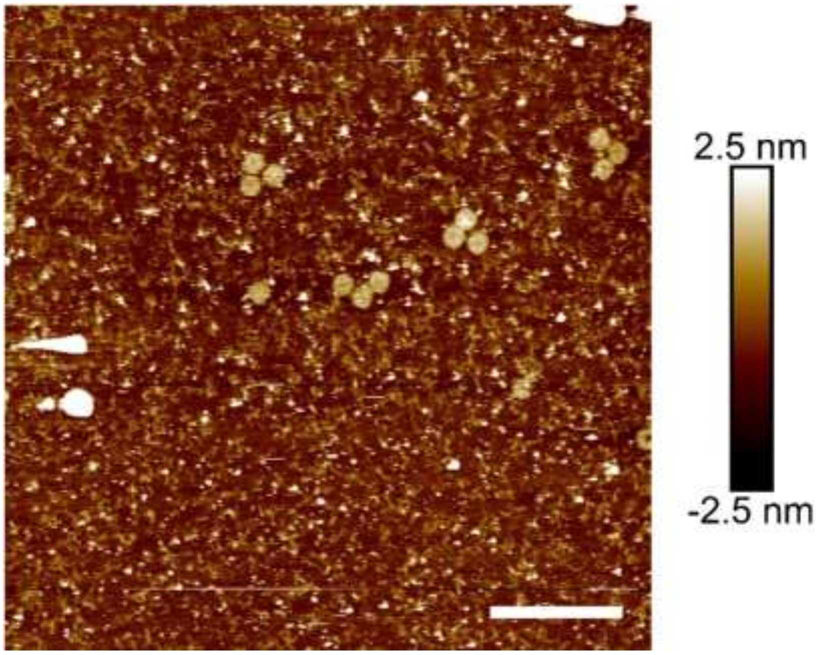

Supplementary Figure 56. AFM result of L-shaped trimer. Scale bar: 600 nm.

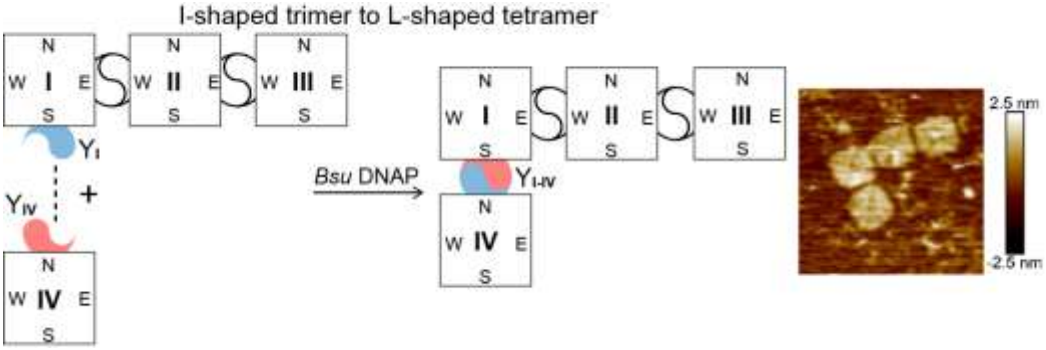

Supplementary Figure 57. Schematic of rearrangement control in origami. The I-shaped trimer was combined with a single origami square to form an L-shaped tetramer. Units I, II and III performed an I-shaped trimer with working site Y_I in the south side of unit I, and the corresponding working site Y_{IV} in the north side of unit IV. The *Bsu* DNA polymerase large fragment drives the combination of trimer and unit IV into an L-shaped tetramer. No cut sites were designed between units II and III, so the I-shaped trimer remained its shape.

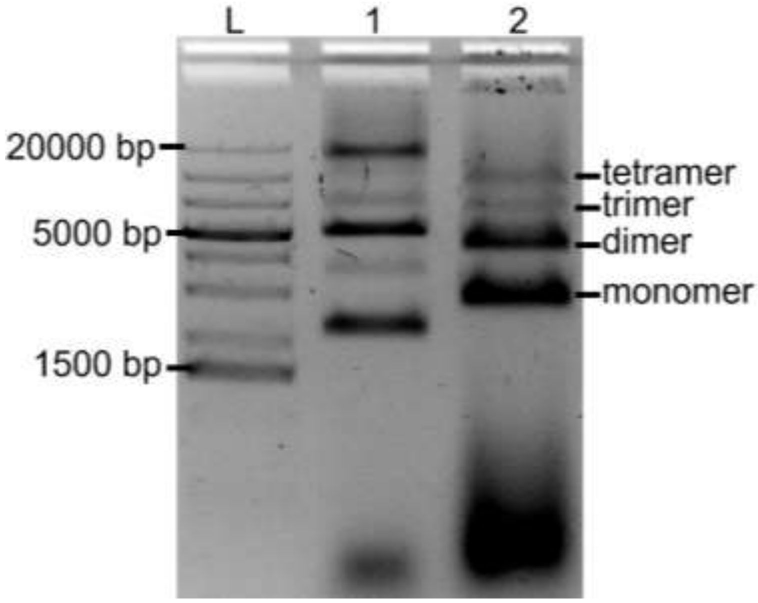

Supplementary Figure 58. Native polyacrylamide gel electrophoresis results of the O-shaped tetramer. Lane L: 1 kb+ ladder. Lane 1: the I-shaped trimer. Lane 2: the I-shaped trimer was converted and mixed with an extra unit into an O-shaped tetramer by *Bsu* DNAP with dTTP/dGTP/dCTP addition. The yield of the O-shaped tetramer is only 3% (*N*=3). The origami units were mixed without gel purification, thus excess unformed staple strands existed at the bottom of the gel.

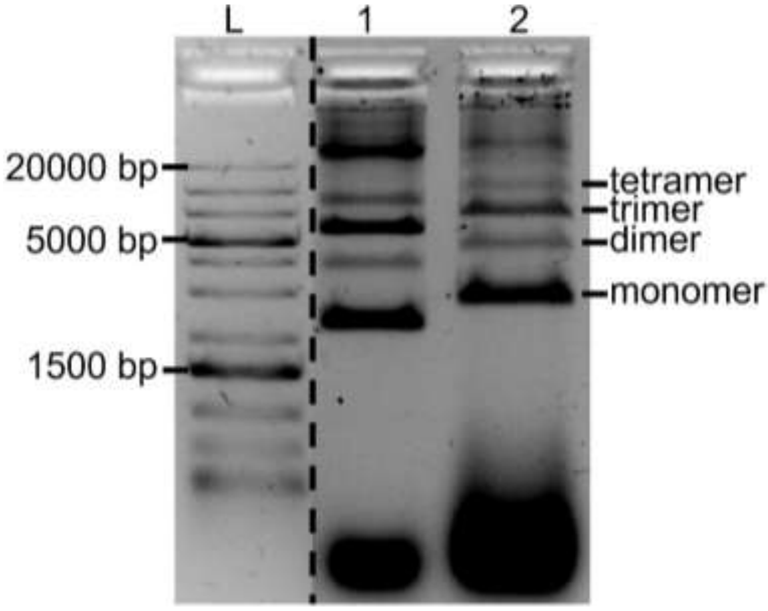

Supplementary Figure 59. Native polyacrylamide gel electrophoresis results of the L-shaped tetramer. Lane L: 1 kb+ ladder. Lane 1: the I-shaped trimer. Lane 2: the I-shaped trimer was rearranged with an extra unit IV into an L-shaped tetramer by *Bsu* DNAP with dTTP/dGTP/dCTP addition. The yield of the L-shaped tetramer is only 10% (*N*=3). The origami units were mixed without gel purification, thus excess unformed staple strands existed at the bottom of the gel.

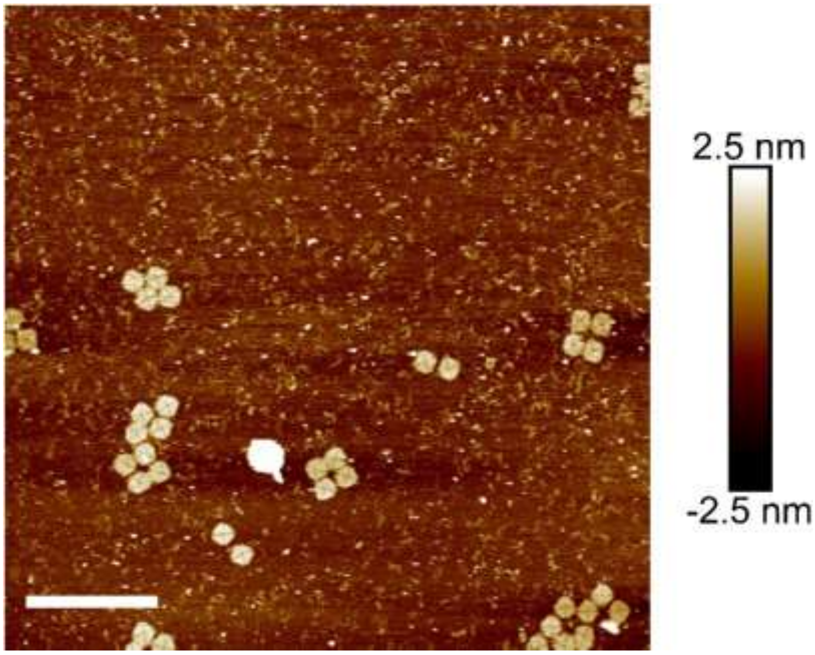

Supplementary Figure 60. AFM result of O-shaped tetramer. Scale bar: 600 nm.

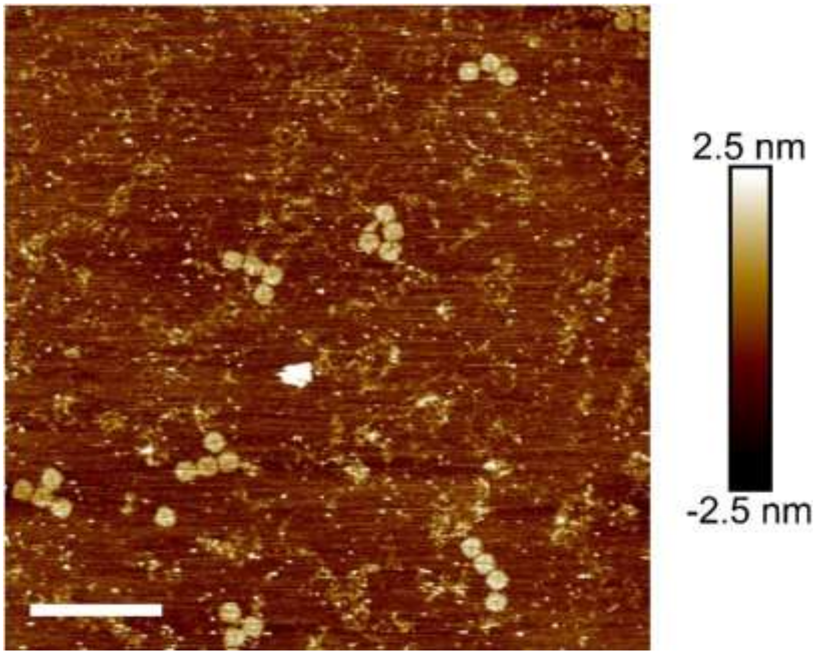

Supplementary Figure 61. AFM result of L-shaped tetramer. Scale bar: 600 nm.

Supplementary Table

Supplementary Table 1. Stacking free energy parameters at the nick sites of DNA[2]. Two base-pairs flanking the nick site with different sequence compositions. KL, dinucleotide stacks. The loss of stacking force between the base-pair flanking the nick induces a kink in the DNA duplex resulting in end breathing and branch migration.

**KL (kcal mol^{-1})**	**A**	**T**	**G**	**C**
**A**	-1.11	-1.34	-1.06	-1.81
**T**	-0.19	-1.11	-0.55	-1.43
**G**	-1.43	-1.81	-1.44	-2.17
**C**	-0.55	-1.06	-0.91	-1.44
**Average: -1.21**				

Supplementary References

1 Zadeh, J. N., Steenberg, C. D., Bois, J. S., Wolfe, B. R., Pierce, M. B., Khan, A. R., Dirks, R. M., & Pierce, N. A. NUPACK: Analysis and design of nucleic acid systems. *J. Comput. Chem.* **32**, 170–173 (2011).

2 Protozanova, E., Yakovchuk, P. & Frank-Kamenetskii, M. D. Stacked–Unstacked Equilibrium at the Nick Site of DNA. *J. Mol. Biol.* **342**, 775-785 (2004).
