# Supplementary material for: Synthetic molecular switches driven by DNA-modifying enzymes
Source: Nat Commun. 2024 May 6;15:3781. doi: 10.1038/s41467-024-47742-2 (PMC11074287; doi:10.1038/s41467-024-47742-2)
Supplement: Supplementary file 3 — Description of Additional Supplementary Files [file 41467_2024_47742_MOESM3_ESM.pdf]

Description of Additional Supplementary Files

File Name: Supplementary Data 1

Description: all DNA sequences used in this work were shown in with type, name, and sequence, and used in structural parts. The “Minimalist systems” sheet includes DNA strands for the simplest systems and the comparison between enzymatic- and toehold-mediated strand displacement. Strands of types X and Y in 5×5 lattice structures were illustrated in sheets “SST 5×5 Square X switch strands” and “SST 5×5 Square Y switch strands”. The “SST 8×8 Square Core Strands” provided strands for the core region of the 8×8 lattice structure. The cut and paste edges strands for 8×8 lattice in “SST 8×8 Square Edge Strands”. Strands for inside cut sites for 8×8 lattice to form specific shapes are included in “SST 8×8 Square Cut Strands”. Core staple strands to shape a square DNA origami structure are in sheet “Origami 22H Square Core Staples”, and the edge strands for enzymatic treatment are in sheet “Origami 22H Square Edge Staples”.
